# A Phylogenomic Analysis of the Bacterial Phylum Fibrobacteres

**DOI:** 10.3389/fmicb.2015.01469

**Published:** 2016-01-07

**Authors:** Nurdyana Abdul Rahman, Donovan H. Parks, Inka Vanwonterghem, Mark Morrison, Gene W. Tyson, Philip Hugenholtz

**Affiliations:** ^1^Australian Centre for Ecogenomics, School of Chemistry and Molecular Biosciences, The University of QueenslandBrisbane, QLD, Australia; ^2^Advanced Water Management Center, The University of QueenslandBrisbane, QLD, Australia; ^3^Microbial Biology and Metagenomics, The University of Queensland Diamantina Institute, Translational Research InstituteBrisbane, QLD, Australia; ^4^Genomics and Computational Biology, Institute for Molecular Bioscience, The University of QueenslandBrisbane, QLD, Australia

**Keywords:** fibrobacteres, TG3, termite gut, anaerobic digester, comparative genomics

## Abstract

The Fibrobacteres has been recognized as a bacterial phylum for over a decade, but little is known about the group beyond its environmental distribution, and characterization of its sole cultured representative genus, *Fibrobacter*, after which the phylum was named. Based on these incomplete data, it is thought that cellulose hydrolysis, anaerobic metabolism, and lack of motility are unifying features of the phylum. There are also contradicting views as to whether an uncultured sister lineage, candidate phylum TG3, should be included in the Fibrobacteres. Recently, chitin-degrading cultured representatives of TG3 were isolated from a hypersaline soda lake, and the genome of one species, *Chitinivibrio alkaliphilus*, sequenced and described in detail. Here, we performed a comparative analysis of *Fibrobacter succinogenes, C. alkaliphilus* and eight near or substantially complete Fibrobacteres/TG3 genomes of environmental populations recovered from termite gut, anaerobic digester, and sheep rumen metagenomes. We propose that TG3 should be amalgamated with the Fibrobacteres phylum based on robust monophyly of the two lineages and shared character traits. Polymer hydrolysis, using a distinctive set of glycoside hydrolases and binding domains, appears to be a prominent feature of members of the Fibrobacteres. Not all members of this phylum are strictly anaerobic as some termite gut Fibrobacteres have respiratory chains adapted to the microaerophilic conditions found in this habitat. Contrary to expectations, flagella-based motility is predicted to be an ancestral and common trait in this phylum and has only recently been lost in *F. succinogenes* and its relatives based on phylogenetic distribution of flagellar genes. Our findings extend current understanding of the Fibrobacteres and provide an improved basis for further investigation of this phylum.

## Introduction

The phylum Fibrobacteres is recognized as a major line of descent in the bacterial domain but is understudied due to limited representation by axenic cultures. The only described genus in this lineage is *Fibrobacter* (Montgomery et al., 1988, orginally classified as *Bacteroides* Hungate, 1950), after which the phylum was named (Ludwig and Klenk, 2001). *Fibrobacter* currently comprises two species, *Fibrobacter succinogenes* isolated from a cow rumen (Hungate, 1950) and *Fibrobacter intestinalis* isolated from a rat cecum (Montgomery and Macy, 1982), of which the former has a publicly available genome sequence (Suen et al., 2011). Both species are primary degraders of cellulosic plant biomass in herbivore guts (Hungate, 1950; Montgomery et al., 1988), which has prompted the suggestion that cellulose degradation may be a unifying feature of the phylum (Ransom-Jones et al., 2012, 2014). This is supported by culture-independent 16S rRNA-based environmental surveys identifying relatively high numbers of diverse members of the Fibrobacteres in cellulolytic ecosystems (Ransom-Jones et al., 2012, 2014).

Candidate phylum TG3 (Termite group 3) is often phylogenetically associated with the Fibrobacteres based on comparative analyses of the 16S rRNA gene (Hongoh et al., 2005, 2006; Warnecke et al., 2007; He et al., 2013; Sorokin et al., 2014). TG3 was initially detected in environmental surveys of termite guts, but was later found to be present in a diverse range of habitats (Hongoh et al., 2005). Recently, the first isolates for TG3 have been described (Sorokin et al., 2012), one of which has been named *Chitinivibrio alkaliphilus* and its genome sequenced (Sorokin et al., 2014). *C. alkaliphilus* is a haloalkaliphilic anaerobic chitin-utilizing bacterium isolated from soda lake sediments. There have been conflicting views as to whether TG3 should be merged with the Fibrobacteres or retained as a separate phylum (Sorokin et al., 2014).

Recent developments in metagenomics provide the opportunity to obtain genomic representation of uncultured Fibrobacteres and TG3 populations which can be used to evaluate conservation of polymer (cellulose and chitin) degradation and other metabolic properties across these lineages, and the robustness of the association between the two phyla. Here, we used differential coverage binning (Albertsen et al., 2013) to obtain seven Fibrobacteres and one TG3 population genomes from termite gut, sheep rumen and anaerobic digester samples. This substantially expands the genomic coverage of both groups and comparative analyses of these genomes with the publicly available *F. succinogenes* and *C. alkaliphilus* genomes suggest that polymer hydrolysis is a phylogenetically widespread trait in these lineages. We propose that candidate phylum TG3 should be classified as part of the Fibrobacteres based on shared character traits and phylogenetic analyses of concatenated gene sets supporting a robust association between the two groups.

## Materials and methods

### Samples and metagenome sequencing

DNA samples described in previous 16S rRNA community profiling studies were used in the present study for shotgun sequencing. These comprised four termite samples; MC05, MC06, MC07, and IN01 (Abdul Rahman et al., 2015) and six anaerobic digester samples taken from 3 reactors (AD1-3) at two time points (day 96 and 362; Vanwonterghem et al., 2014). A publicly available sheep rumen metagenome (BioProject acc. PRJNA214227) was also included in the study together with two reference genomes; *F. succinogenes* S85 (BioProject acc. PRJNA41169) and *C. alkaliphilus* ACht1 (BioProject acc. PRJNA195589). Shotgun libraries were prepared using the Nextera XT Sample Preparation Kit (or TruSeq DNA Sample Preparation Kits v2 for AD1-3 day 96) (Illumina, San Diego, CA, USA) and library DNA concentrations were measured using the QuantIT kit (Molecular probes, Carsbad, CA, USA) and equimolar-pooled for sequencing. Between a quarter and a third of an Illumina HiSeq 2000 flowcell of paired-end sequences (2 × 100 bp with an average fragment size of 320) were obtained for each library.

### Sequence assembly and population genome binning

For the termite datasets, paired-end reads were merged and adaptors removed using SeqPrep v1.1 (https://github.com/jstjohn/SeqPrep), and then quality trimmed with a Q-value of 20 using Nesoni v0.128 (http://www.vicbioinformatics.com/software.nesoni.shtml). Adaptor removal and quality trimming was performed using CLC Workbench v6 (CLC Bio, Taipei, Taiwan) for the anaerobic digester (AD) datasets. *De novo* assemblies of the termite and AD datasets were generated using CLC Workbench v6 using a word size of 63 and a minimum contig length of ≥500 bp. Reads from each sample were mapped to the assembled contigs using the BWA-MEM algorithm in BWA v0.5.5 with default parameters (Li, 2013). Population genomes were obtained using the differential coverage binning method of GroopM (Imelfort et al., 2014) with default parameters. The termite and AD metagenomes were binned independently using GroopM v0.1 and v0.2, respectively. Briefly, reads from each sample were mapped onto their corresponding co-assemblies and coverage patterns for each scaffold were calculated, transformed, and projected onto a 3-dimensional space in which scaffolds from the same population genome cluster together (Imelfort et al., 2014). Manual refinement of selected genomes was performed using the GroopM refine function in order to merge bins with compatible genome characteristics (i.e., GC and coverage statistics) and split bins that appeared to be aggregates of two or more genomes. For the sheep rumen metagenome, population genomes were recovered using a distribution-based binning method (DBB v1.0.1; https://github.com/dparks1134/DBB) since multiple related samples were not available for differential coverage binning. This method identified contigs likely to belong to the same population based on the GC-content, tetranucleotide signature, and coverage of individual contigs. Genome completeness and contamination was estimated using lineage-specific marker sets determined by CheckM v1.0.3 (Parks et al., 2015).

### Taxonomic assignment of population genomes

Population genomes estimated to be >60% complete and <10% contaminated were placed in a maximum likelihood tree of 2358 reference genomes based on a concatenation of 83 marker genes as described previously (Soo et al., 2014). The inferred phylogeny was used to identify putative members of the Fibrobacteres and TG3 lineages. To corroborate genome-based identifications, 16S rRNA genes or gene fragments associated within the population genomes were identified with CheckM (Parks et al., 2015) and aligned with reference Fibrobacteres and TG3 sequences obtained from SILVA database release 119 (Quast et al., 2012) using ssu-align v0.1 (Nawrocki et al., 2009). Poorly represented leading and trailing columns of the multiple sequence alignment were manually trimmed, and a maximum likelihood tree inferred with FastTree v2.1.7 (Price et al., 2009). Sequences greater than 1200 nt were selected for the purposes of calculating non-parametric bootstrap support values. These selected sequences were reanalyzed using FastTree followed by 100 bootstrap replicates, and support values propagated to the full tree consisting of both short and long sequences. Phylogenetic tree and bootstraps values were scaled and edited in ARB (Ludwig et al., 2004) and Adobe Illustrator CS6 (Adobe). All Fibrobacteres/TG3 population genomes have been deposited at JGI IMG/ER under the accessions 2522572000, 2522572002, 2522572004, 2522572005, 2582580742, 2582580743, 2585427501, 2606217802, and GenBank/DDBJ/EMBL as individual Biosamples under the multispecies BioProject PRJNA293241.

### Genome annotation and metabolic reconstruction

The draft Fibrobacteres and TG3 genomes were uploaded to the Integrated Microbial Genomes with Microbiome Samples-Expert Review (IMG/ER) system (Markowitz et al., 2014) for automated annotation with IMG/M Metagenome Gene Calling. KEGG pathway maps were visualized by uploading KEGG (Kyoto Encyclopedia of Genes and Genomes) annotations to the KEGG Mapper—Color Pathway (http://www.genome.jp/kegg/tool/map_pathway3.html). Glycoside hydrolases (GHs) and carbohydrate-binding modules (CBMs) were identified using the CAZy database (Lombard et al., 2014) via dbCAN (Yin et al., 2012). Signal peptide predictions were performed using SignalP (Petersen et al., 2011). IMG/ER identified methyl-accepting proteins were scanned for chemotaxis protein domain using InterProScan5 (Jones et al., 2014). The draft genomes were also annotated with PROKKA v1.7 using default settings (Seemann, 2014). The final gene and pathway inventories of the putative Fibrobacteres and TG3 genomes were based on a combination of the IMG and PROKKA annotations and functional classifications based on COG (Clusters of Orthologous Groups), KO, Enzyme, Pfam, and TIGRfam assignments. Metabolic reconstructions based on these inventories were prepared in Adobe Illustrator CS6 (Adobe).

### Genome and protein family comparative analyses

Average amino acid identities (AAI) between homologs in genome pairs were calculated using the AAI calculator with default settings in CompareM v0.0.4 (https://github.com/dparks1134/CompareM). Heat maps of the relative abundance of genes and pathways within genomes were generated with STAMP v2.0.9 (Parks et al., 2014). Phylogenetic analysis of selected proteins (GHs, CBMs, cytochrome bd, fibro-slime domain, flagellar proteins) in the population genomes was performed by identifying homologs within IMG v4.510 (Markowitz et al., 2014) using BLASTP. A gene was considered homologous if it had an expectation value ≤1e-5, an amino acid identity ≥50%, and an alignment length of ≥30%. Proteins alignments were obtained using MAFFT v7.221 (Standley, 2013) and trees inferred using FastTree v2.1.7 under the WAG+G models and support values determined using 100 non-parametric bootstrap replicates.

## Results and discussion

### Recovery of population genomes from environmental metagenomic datasets

**Figure 1:**
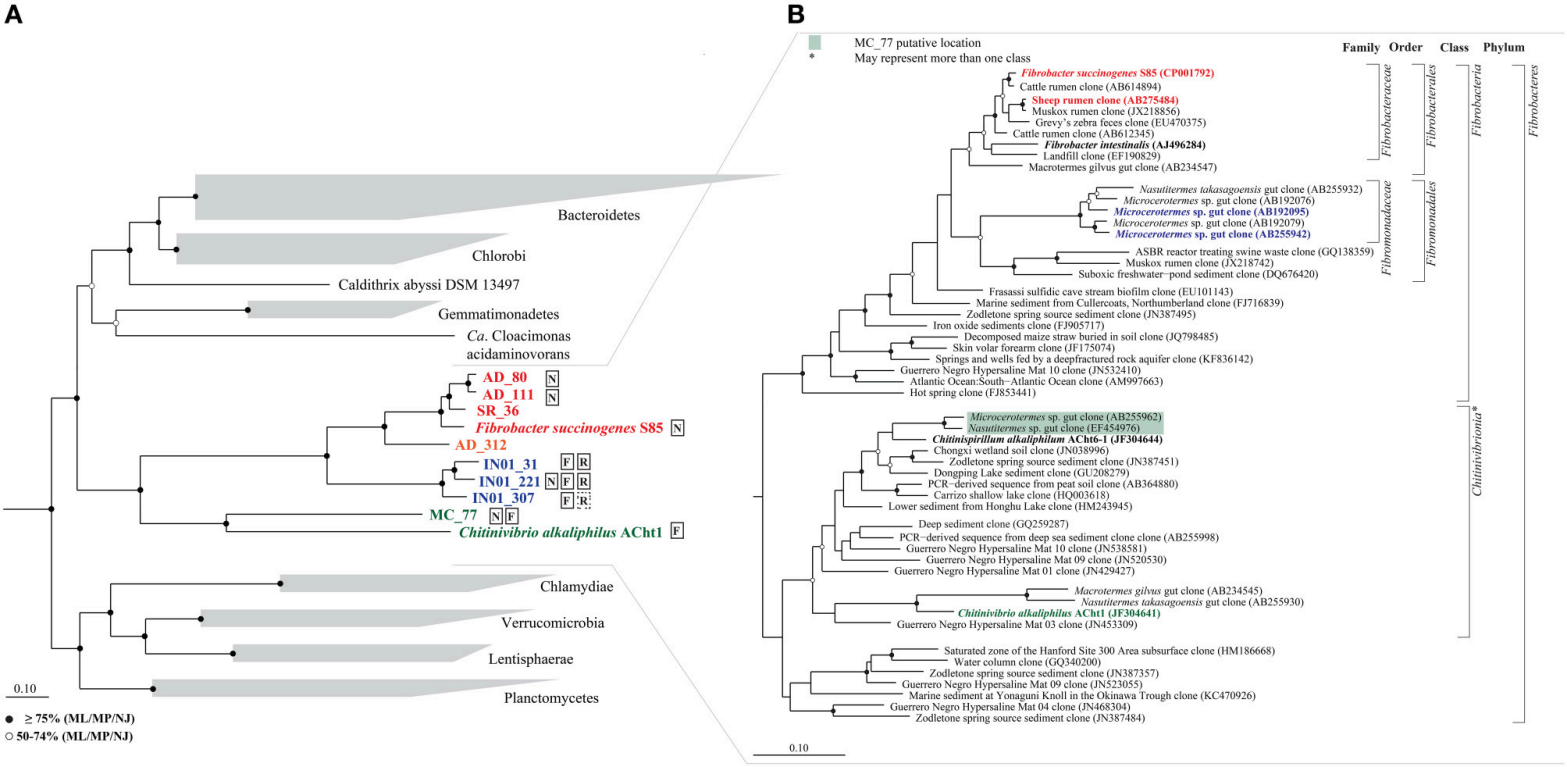
**Phylogenetic analysis of the phylum Fibrobacteres**. **(A)** Maximum likelihood tree of the phylum Fibrobacteres based on alignment of 83 concatenated proteins as previously described (Soo et al., 2014). The tree was inferred using an outgroup comprising 2358 genomes from 33 phyla. For clarity, only the immediate phylum-level neighborhood of the Fibrobacteres is shown. *Fibrobacteraceae* genomes are shown in red; *Fibromonadaceae* in blue; and *Chitinivibronia* in green. Fibrobacteres genomes encoding nitrogen-fixing, flagellar and/or respiratory genes are indicated by N, F, and R in boxes (dotted box indicates incomplete genes), respectively. Bootstrap support for interior nodes using multiple inference methods is shown according to the legend at the lower left of the figure; ML, Maximum Likelihood; MP, Maximum Parsimony; NJ, Neighbor Joining. **(B)** Maximum likelihood tree based on 16S rRNA genes from Fibrobacteres and TG3 obtained from SILVA database release 119 (Quast et al., 2012). The closest matches to the partial 16S rRNA sequences obtained from the population genomes are indicated by color matching to **(A)**, noting that the position of MC_77 is estimated since this genome lacks a 16S rRNA sequence. Isolates are bolded in black. Taxonomic group names by rank are proposed to the right of the tree, also see main text. Node support values are as described for **(A)**.

Bulk DNAs extracted from termite whole gut samples for 16S rRNA-based community profiling (Abdul Rahman et al., 2015) were used in the present study. A total of 74 Gb of Illumina 2 × 100 bp data were sequenced from four sets of *Microcerotermes* whole gut samples (30 guts per set) obtained from the same nest, IN01, in Brisbane, Queensland. Similarly, 71 Gb was sequenced from three sets of *Nasutitermes* whole gut samples (30 guts per set) collected from three mounds within a 1 km radius in Murphy's Creek, South East Queensland (MC05, MC06, MC07). Bulk DNAs extracted from three lab-scale anaerobic digesters collected at two timepoints [AD1 to 3; reported in Vanwonterghem et al. (2014)] were also sequenced to produce a total of 111 Gb (2 × 100 bp Illumina reads). Publicly deposited metagenomic datasets were also screened for the presence of Fibrobacteres genomes (data not shown), of which one, a sheep rumen microbiome (BioProject acc. PRJNA214227, SRR948090; 9.9 Gb of 2 × 100 bp Illumina reads) produced a genome of sufficient quality for comparative analysis. Sequence datasets from each habitat were independently assembled and binned (Supplementary Table 1). A total of 303 population genomes with >60% completeness and <10% contamination (estimated by CheckM; Parks et al., 2015) were obtained from the four sample types and, of these, eight were phylogenetically affiliated with the publicly available isolate genomes of Fibrobacteres (*F. succinogenes* S85, acc. PRJNA41169) and TG3 (*C. alkaliphilus* ACht1, acc. PRJNA195589; Figure 1). All eight genomes had low contamination, four were near complete and four were substantially complete according to CheckM estimates and classification (Parks et al., 2015). Together with the two reference organisms, genome size, and GC content range from 2.4 to 3.8 Mb and 37.4 to 53.9%, respectively (Table 1) comparable to other phyla of similar phylogenetic breadth (Lightfield et al., 2011).

**Table 1:** **Summary statistics of Fibrobacteres isolate and population genomes**.

**Genomes**	**Source**	**Estimated population genome size (Mb)**	**GC (%)**	**No. of contigs**	**Estimated Completeness^a^ (%)**	**Estimated Contamination^a^ (%)**	**No. of genes**	**rRNA genes**	**Coding Density (%)**	**References**
**PURE CULTURE**										
*Fibrobacter succinogenes* S85	Cow rumen	3.8	48.1	1	100.0	2.5	3188	5S,16S,23S	91.3	Suen et al., 2011
*Chitinivibrio alkaliphilus*ACht1	Soda Lake	2.6	46.2	99	97.9	0.0	2346	5S,16S,23S	93.1	Sorokin et al., 2014
**POPULATION GENOME**										
AD_80	Bioreactor	3.3	51.4	123	88.9	1.7	2801	5S	91.1	Present study
AD_111	Bioreactor	3.6	50.2	189	88.1	0.2	3069	5S,16S,23S	91.3	Present study
SR_36	Sheep rumen	3.4	53.9	50	99.2	1.7	2906	5S	93.1	PRJNA214227^b^
AD_312	Bioreactor	2.8	37.4	56	100.0	3.5	2392	–	90.9	Present study
IN01_31	*Microcerotermes* gut	3.2	43.2	220	96.6	2.6	3417	5S,23S	88.5	Present study
*Fibromonas termitidis*^c^	*Microcerotermes* gut	3.2	43.1	211	98.3	1.7	3355	16S	90.7	Present study
IN01_307	*Microcerotermes* gut	2.6	41.5	157	87.9	1.7	2779	–	92.4	Present study
MC_77	*Nasutitermes* gut	2.4	52.4	317	73.3	0.0	2324	–	85.2	Present study

a *Estimated completeness and contamination based on lineage-specific single copy marker genes (Parks et al., 2015)*.

b *Bioproject accession number*.

c *IN01_221*.

### An expanded phylogenetic classification of the phylum fibrobacteres

We constructed a phylogenetic tree based on a concatenated alignment of 83 bacterial single copy marker genes (Dupont et al., 2012). The ingroup comprised the two complete reference genomes representing the Fibrobacteres (Suen et al., 2011) and TG3 (Sorokin et al., 2014) lineages and eight population genomes obtained in this study (Table 1). We evaluated the monophyly of these genomes using an outgroup consisting of 2358 genomes from 33 phyla. The Fibrobacteres and TG3 genomes formed a robustly monophyletic group (Figure 1A) supporting the previously noted relationship between these lineages (Hongoh et al., 2006; Warnecke et al., 2007; Krieg et al., 2011; He et al., 2013; Mikaelyan et al., 2015). Therefore, we propose to amalgamate TG3 as one or more classes within the phylum Fibrobacteres based on this robust phylogenetic association and shared character traits described below. Additionally, all 10 ingroup genomes contain signature inserts in their RNA polymerase β' subunit and serine hydroxymethyltransferase genes that identify them as members of the FCB superphylum (Gupta, 2004). Using the partial 16S rRNA gene sequences identified in a number of the population genomes (Table 1), we placed the genomes in the broader context of the 16S rRNA-defined Fibrobacteres lineage (Figure 1B). *F. succinogenes* S85, AD_80, AD_111 and SR_36 belong to the family *Fibrobacteraceae* (Spain et al., 2010), with AD_312 likely representing a separate family in the same order (*Fibrobacterales*) based on AAI similarities (Supplementary Table 2; Konstantinidis and Tiedje, 2005). IN01_31, IN01_221, and IN01_307 form a monophyletic cluster found exclusively in termite guts previously referred to as candidate order TFG-1 (Warnecke et al., 2007). We propose the candidatus name, *Fibromonas termitidis*, for the most complete of these genomes, IN01_221, and the family and order names, *Fibromonadaceae* and *Fibromonadales* for this group and related 16S rRNA sequences (Figure 1). Unfortunately, population genome MC_77 lacked a 16S rRNA sequence so could not be placed within the 16S framework. However, it likely belongs to the TG3 lineage, and more specifically in the termite cluster proximate to isolate ACht6-1 (Figure 1; Sorokin et al., 2012). Sorokin et al. (2014) proposed the class *Chitinivibrionia* to accommodate *C. alkaliphilus* ACht1, which now becomes the second recognized class within the Fibrobacteres due to its amalgamation with TG3 (Figure 1). We have provisionally included MC_77 in the class *Chitinivibrionia*, however, given the depth of the relationship with *C. alkaliphilus* (Figure 1), MC_77 and isolate ACht6-1 may represent a distinct class within the expanded phylogenetic representation of the Fibrobacteres phylum.

### Inferred metabolism of fibrobacteres genomes

We performed comparative analyses of the two isolate and eight draft population genomes (Table 1) to infer metabolic properties associated with the *Fibrobacteres* in the context of their environmental settings.

#### Polymer hydrolysis

##### Cellulases

**Figure 2:**
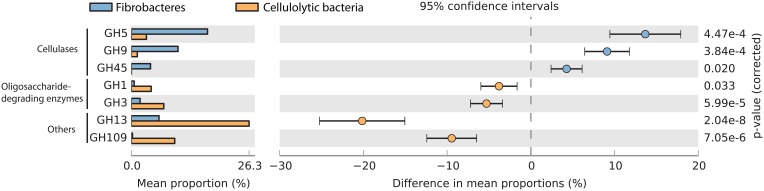
**Glycoside hydrolase families with a significant difference in mean proportions ≥1% between Fibrobacteres and other cellulolytic bacteria and a *p* ≤ 0.05**. Statistical significance was assessed using Welch's *t*-test with Bonferroni multiple test correction.

Members of the Fibrobacteres are best known for their ability to hydrolyze plant polymers in anoxic habitats such as the bovine rumen (Suen et al., 2011; Jewell et al., 2013; Ransom-Jones et al., 2014) and termite gut (Warnecke et al., 2007; He et al., 2013). Therefore, we began by identifying genes encoding glycoside hydrolases (GHs) classified according to the CAZy database (Lombard et al., 2014). All 10 genomes contained numerous GHs representing between 1.2 and 3.5% of the total genes, which is higher than the bacterial average of 0.9%, but similar to other cellulolytic bacteria (2%; Table 2). However, polymer-degrading enzymes are highly over-represented in the Fibrobacteres GH inventory relative to other recognized cellulolytic bacteria (cellulases—25 vs. 3%, xylanases 15 vs. 4%). The proportion of Fibrobacteres GHs with signal peptides is also much higher than that for the average Fibrobacteres gene (28.6 vs. 7.4, respectively) which is as expected for proteins involved in extracellular deconstruction of carbohydrate polymers (Lombard et al., 2014). A quarter of the GHs in the *Fibrobacteraceae* and *Chitinivibrionia* and over a third of the GHs in the *Fibromonadaceae* are cellulases. Most of the cellulases belong to families GH5 and GH9 which are widely distributed in bacteria (Figure 2; present in ≥50% of recognized phyla; Table 2; Berlemont and Martiny, 2013). The less common cellulase family GH45, previously noted to be distinctive of *F. succinogenes* (Suen et al., 2011; Dai et al., 2012) and related organisms in the termite hindgut (Warnecke et al., 2007), is present in all studied representatives of the Fibrobacteres, with the exception of *C. alkaliphilus* (Figure 2 and Table 2). Cellulase family GH44 is distinctive of the *Fibrobacteraceae* in the context of the Fibrobacteres although it has been identified in members of six other bacterial phyla. The previously noted absence of the classical exo-acting β-1,4 glucanase families GH6, GH7, and GH48 in *F. succinogenes* is upheld across the phylum supporting the hypothesis that the Fibrobacteres have a distinctive suite of carbohydrate-active enzymes and lignocellulose hydrolysis mechanism (Morrison et al., 2009; Wilson, 2009). Furthermore, the distinctive basic terminal domain (~80 AA in the C-terminus) noted in *F. succinogenes* cellulases (Iyo and Forsberg, 1996; Malburg et al., 1996; Qi et al., 2007, 2008) is widespread in cellulases of all members of the Fibrobacteres. Cellulases play an important role in the habitats from which the Fibrobacteres genomes were obtained (Table 1) with the possible exception of the soda lake from which *C. alkaliphilus* was recovered. Although *C. alkaliphilus* encodes a high proportion of cellulases relative to the bacterial average (Table 2), it was reported to be unable to grow on cellulose as a sole carbon source (Sorokin et al., 2014) indicative of their role being relevant to polymer deconstruction rather than energy acquisition.

**Table 2:** **Inventory of glycoside hydrolases (GHs) identified in the Fibrobacteres genomes, organized by functional category**.

**CAZy family**	**Known activity**	**pfam domain**	**Fibrobacteria**								**Chitinivibrionia**		**Cellulolytic bacteria (35 genomes^a^)**	**Bacterial average across 2038 genomes**.		
			**Fibrobacteraceae**					**Fibromonadaceae**			***C. alkali- philus* ACht1**			**Avg**	**Prevalence (%)**	**No. of phyla^b^**
			***F. succino-genes* S85**	**AD_80**	**AD_ 111**	**SR_ 36**	**AD_ 312**	**IN01_31**	**IN01_221**	**IN01_307**		**MC_ 77**	**Avg**			
**CELLULASES**																
GH5	Cellulase	PF00150	13.0	16.7	16.7	15.2	18.8	27.5	18.0	15.2	6.3	23.3	2.37	1.46	30.9	17
GH9	Endoglucanase	PF00759	9.0	10.3	10.4	8.7	5.8	10.0	12.8	12.1	18.8	6.7	0.91	0.31	9.6	15
GH44	Endoglucanase	PF12891	1.0	1.3	1.0	1.1	1.5	–	–	–	–	–	0.03	0.03	2.3	7
GH45	Endoglucanase	PF02015	4.0	3.9	5.2	4.4	2.9	2.5	7.7	9.1	–	3.3	0.02	0.01	0.3	3
Subtotals (%)			27.0	32.1	33.3	29.4	29.0	40.0	38.5	36.4	25.0	33.3	3.33	1.81		
**CHITINASES**																
GH18	Chitinase	PF00704	2.0	2.6	2.1	2.2	2.9	2.5	2.6	3.0	3.1	3.3	3.12	2.16	30.0	19
GH19	Chitinase	PF00182	–	–	–	–	–	–	–	–	6.3	–	0.55	0.32	9.7	10
GH20	β-hexosaminidase	PF00728	–	–	–	–	–	–	–	–	–	3.3	1.15	0.91	23.2	17
Subtotals (%)			2.0	2.6	2.1	2.2	2.9	2.5	2.6	3.0	9.4	6.7	4.82	3.39		
**HEMICELLULASES**																
GH8	Endo-xylanases	PF01270	6.0	3.9	7.3	7.6	5.8	5.0	5.1	3.0	15.6	3.3	0.81	0.56	20.1	17
GH10	Endo-1,4-β-Xylanase	PF00331	8.0	5.1	7.3	6.5	2.9	2.5	2.6	3.0	6.3	–	1.06	0.43	12.7	14
GH11	Xylanase	PF00457	4.0	1.3	4.2	5.4	1.5	2.5	5.1	3.0	–	–	0.31	0.08	4.3	11
GH26	β-mannanase and xylanase	PF02156	4.0	3.9	4.2	3.3	2.9	–	–	3.0	–	–	0.98	0.16	6.9	11
GH53	Endo-1,4-β-xylanase	PF07745	2.0	1.3	1.0	2.2	1.5	2.5	2.6	3.0	–	–	0.55	0.22	10.6	14
Subtotals (%)			24.0	15.4	21.0	25.0	14.5	12.5	15.4	15.2	21.9	3.3	3.72	1.45		
**DEBRANCHING ENZYMES**																
GH16	Xyloglucanases and xyloglycosyltransferases	PF00722	4.0	5.1	4.2	3.3	4.4	0.0	0.0	0.0	3.1	0.0	1.72	0.78	19.7	20
GH74	Endoglucanases and oglucanases	–	2.0	3.9	2.1	2.2	2.9	0.0	0.0	0.0	0.0	0.0	0.27	0.10	4.3	9
GH51	α-L-arabinofuranosidase	–	1.0	1.3	1.0	1.1	1.5	0.0	2.6	3.0	0.0	0.0	0.78	0.41	15.0	14
GH54	α-L-arabinofuranosidase	PF09206	1.0	0.0	1.0	0.0	0.0	0.0	0.0	0.0	0.0	0.0	0.00	0.02	0.9	6
Subtotals (%)			8.0	10.3	8.3	6.5	8.7	0.0	2.6	3.0	3.1	0.0	2.77	1.31		
**OLIGOSACCHARIDE-DEGRADING ENZYMES**																
GH1	β-glucosidase and other β-linked dimers	PF00232	0.0	0.0	0.0	0.0	0.0	0.0	0.0	0.0	3.1	3.3	2.73	3.66	46.6	17
GH2	β-galactosidases and other β–linked dimers	PF00703 PF02836 PF02837	2.0	2.6	2.1	2.2	2.9	0.0	0.0	3.0	3.1	0.0	2.88	1.92	38.2	18
GH3	Mainly β-glucosidases	PF00933 PF01915	3.0	2.6	2.1	1.1	2.9	5.0	0.0	3.0	0.0	0.0	4.68	5.87	73.4	27
GH43	Arabinases and xylosidases	PF04616	14.0	11.5	9.4	13.0	13.0	5.0	5.1	0.0	0.0	3.3	2.82	1.30	22.2	15
GH52	β-xylosidase	PF03512	0.0	0.0	0.0	0.0	0.0	0.0	0.0	0.0	0.0	3.3	0.04	0.03	1.5	5
Subtotals (%)			19.0	16.7	13.5	16.3	18.8	10.0	5.1	6.1	6.2	10.0	13.14	12.79		
**CELLOBIOSE/CHITOBIOSE PHOSPHORYLASE**																
GH84	Cellobiose/chitobiose phosphorylase	PF06165 PF06205	0.0	0.0	0.0	0.0	0.0	0.0	0.0	0.0	3.1	0.0	0.23	0.16	4.8	7
GH94	Cellobiose/chitobiose phosphorylase	PF06165 PF06205	1.0	1.3	1.0	1.1	1.5	5.0	2.6	3.0	6.3	3.3	0.58	0.69	18.8	18
Subtotals (%)			1.0	1.3	1.0	1.1	1.5	5.0	2.6	3.0	9.4	3.3	0.81	0.85		
**OTHERS**																
GH13	Unknown	PF02903	3.0	3.9	3.1	3.3	5.8	7.5	10.3	12.1	6.3	6.7	16.52	16.67	80.2	27
GH23	Lysozyme type G/peptidoglycan lyase/chitinase	–	3.0	3.9	3.1	3.3	4.4	12.5	10.3	12.1	12.5	20.0	8.01	17.21	80.7	30
GH27	Unknown		1.0	1.3	1.0	1.1	1.5	0.0	0.0	0.0	0.0	0.0	0.44	0.09	5.1	10
GH30	Glucosylceramidase	PF02055	4.0	3.9	4.2	4.4	2.9	0.0	2.6	0.0	0.0	0.0	0.52	0.31	11.6	15
GH57	α-amylase	PF03065	3.0	3.9	2.1	2.2	2.9	5.0	2.6	3.0	3.1	10.0	0.70	2.43	22.8	27
GH77	Amylomaltase or 4-α-glucanotransferase	PF02446	1.0	1.3	1.0	1.1	1.5	2.5	2.6	3.0	3.1	3.3	1.47	3.20	55.0	26
GH95	α-1,2-L-fucosidase/α-L-fucosidase	–	1.0	1.3	1.0	1.1	1.5	0.0	0.0	0.0	0.0	0.0	0.35	0.22	9.8	14
GH105	Unknown	PF07470	1.0	0.0	0.0	0.0	0.0	0.0	0.0	0.0	0.0	0.0	0.77	0.31	12.2	14
GH106	α-L-rhamnosidase	–	0.0	0.0	0.0	0.0	0.0	0.0	0.0	0.0	0.0	3.3	0.16	0.09	4.5	8
GH109	α-N-acetylgalactosaminidase	–	0.0	0.0	0.0	1.1	1.5	0.0	0.0	0.0	0.0	0.0	5.81	5.61	65.1	27
GH116	Unknown		1.0	1.3	1.0	1.1	1.5	2.5	2.6	3.0	0.0	0.0	0.04	0.21	4.7	9
GH127	β-L-arabinofuranosidase	–	1.0	1.3	1.0	1.1	1.5	0.0	2.6	0.0	0.0	0.0	0.77	0.32	14.7	14
Subtotals (%)			19.0	21.8	17.7	19.6	24.7	30.0	33.3	33.3	25.0	43.3	35.51	46.67		
Total estimated glycoside hydrolases			100	78	96	92	69	40	39	33	32	30	–	–		
(Glycoside hydrolases with signal peptide (%))			71.8	64.1	72.1	76.4	48.7	35.6	46.5	40.0	31.4	28.6	–	–		
Total estimated genes			2871	2754	3008	2855	2344	3391	3347	2773	2304	2321	4512^c^	3395^c^		
(Signal peptide to total genes (%))			21.2	17.9	21.6	22.0	15.2	10.8	11.1	11.5	7.4	7.7				
% of genes that are glycoside Hydrolases			3.5	2.8	3.2	3.2	2.9	1.2	1.2	1.2	1.4	1.3	1.2^d^	0.9^d^		

a *Based on Koeck et al. (2014)*.

b *Of a total of 30 bacterial phylum*.

c *Estimated average genes across 35 and 2038 bacterial genomes respectively*.

d *Average of 116 GHs to total genes of 35 and 2038 bacterial genomes respectively*.

##### Hemicellulases and debranching enzymes

As with the cellulases, hemicellulases, and debranching enzymes are present in the Fibrobacteres genomes at much higher relative abundance than the bacterial average, (12.5–32.3 vs. 2.7%) with the exception of MC_77 (3.3%; Table 2). Five hemicellulase families, primarily endoxylanases, were identified in the eight *Fibrobacteria* genomes, while only two were found in the *Chitinivibrionia* (GH8 and GH10), indicating the relative importance of xylan hydrolysis in animal and insect gut ecosystems (Allgaier et al., 2010; Tokuda et al., 2014). Debranching enzymes, responsible for cleaving the side chains (glycosidic and/or ester linkages) from xylan backbones (Sethi and Scharf, 2013), were most prevalent in the *Fibrobacteraceae*. Families GH51 and GH54 are most commonly α-L-arabinofuranosidases responsible for removing arabinose side chains from xylan which is an important constituent of plant lignocellulose (He et al., 2013). GH51s were common in the *Fibrobacteria*, whereas GH54 was only identified in *F. succinogenes* S85 and AD_111 (Table 2), despite being closely related to the AD_80 and SR_36 population genomes (Figure 1).

##### Chitinases

There are three GH families with recognized chitinase activity, GH18, GH19, and GH20, the first two of which are responsible for hydrolysis of insoluble chitin to soluble oligosaccharides in the periplasm (LeCleir et al., 2007; Beier and Bertilsson, 2013). GH20 hydrolyses N-Acetylglucosamine (GlcNAc) molecules from chitin oligomers (Beier and Bertilsson, 2013) or directly from chitin polymers (LeCleir et al., 2007). As expected, *C. alkaliphilus* has the highest proportion of chitinases, approximately three times the bacterial average (Table 2), as it is a chitin-degrading specialist (Sorokin et al., 2014). Furthermore, it has two types of chitinases, GH18 and GH19, which has been postulated to improve substrate degradation due to synergistic enzyme interactions (Beier and Bertilsson, 2013). The closest phylogenetic neighbor of *C. alkaliphilus* in this study, MC_77, similarly has representatives of two chitinase families (GH18 and GH20) and a higher than average proportion of chitinases (Table 2) suggesting that chitin degradation may be occurring in the termite hindgut from which the MC_77 genome was obtained. Chitinases have rarely been considered in the context of Fibrobacteres, however, all representatives of this phylum had GH18-encoding genes at the bacterial average (Table 2), indicating the potential for this function in primarily lignocellulose-degrading gut communities.

##### Accessory attachment genes for polymer degradation

The adhesion of cellulolytic anaerobic bacteria to plant biomass is considered a prerequisite step in breaking down plant cell walls (Morrison and Miron, 2000; Miron et al., 2001). In anaerobic gut bacteria such as *F. succinogenes* and *Ruminococcus* species, surface-associated cellulolytic enzymes complexes (cellulosomes, Dassa et al., 2014), individual GHs possessing non-catalytic carbohydrate-binding modules (CBM, Qi et al., 2005), and Type IV pilin like structures (Pegden et al., 1998) are known to be responsible for adhesion. Fifteen CBM families are represented in the *Fibrobacteria* and *Chitinivibrionia* genomes mostly targeting cellulose, hemicellulose or chitin (Table 3) which is consistent with the GH profiles (Table 2). There are approximately four times as many CBMs in the *Fibrobacteraceae* as in *Fibromonadaceae* and *Chitinivibrionia*, which is also broadly consistent with the relative abundances of GHs in these groups. The CBM families showed lineage-specific patterns. For example both CBM6 and GH35 are all overrepresented in the *Fibrobacteraceae* compared to the *Fibromonadaceae*, but the opposite is apparent for CBM11, CBM32 and CBM50 (Table 3). This suggests that CBMs in the Fibrobacteres have most often been vertically inherited and have not been distributed between lineages by horizontal transfer. This is supported by phylogenetic reconstruction of the Fibrobacteres CBMs which shows mostly vertical transmission and in some lineages expansion of families via gene duplication (Supplementary Figure 1). Higher relative abundances of certain CBM families also correlate with the observed differences in GH family abundances. For example, CBM6 is often associated with the hemicellulose-associated families, GH10 and 43 (Suen et al., 2011), and all three of these families are overrepresented in the *Fibrobacteraceae* relative to the *Fibromonadaceae* (Tables 2, 3).

**Table 3:** **Inventory of accessory attachment genes for polymer hydrolysis identified in the Fibrobacteres genomes, organized by Carbohydrate-binding modules (CBMs)**.

**CAZy family**	**pfam domain**	**Fibrobacteria**								**Chitinivibrionia**		**Cellulolytic bacteria (35 genomes^*a*^)**	**Bacterial average across 2038 sp**.	
		**Fibrobacteraceae**					**Fibromonadaceae**							
		***F. succino-genes* S85**	**AD_80**	**AD_111**	**SR_36**	**AD_312**	**IN01_31**	**IN01_221**	**IN01_307**	***C. alkali- philus* ACht1**	**MC_77**	**Avg**	**Avg**	**Prevalence (%)**
**NON-CATALYTIC CBMS ASSOCIATED WITH: CELLULASES**														
CBM4	pfam02018	8.3	10.9	9.6	8.6	12.2	5.9	6.7	13.3	12.5	15.4	5.6	0.5	5.5
CBM30	–	0.0	0.0	0.0	1.7	0.0	5.9	6.7	0.0	0.0	0.0	0.6	0.1	1.3
CBM51	pfam14498	5.0	4.4	5.8	3.5	6.1	11.8	13.3	6.7	0.0	0.0	0.4	0.4	4.5
Subtotals (%)		13.3	15.2	15.4	13.8	18.4	23.5	26.7	20.0	12.5	15.4	6.6	1.0	
**CHITINASES**														
CBM50	–	8.3	8.7	7.7	8.6	6.1	35.3	20.0	20.0	12.5	30.8	14.6	41.2	76.1
Subtotals (%)		8.3	8.7	7.7	8.6	6.1	35.3	20.0	20.0	12.5	30.8	14.6	41.2	76.1
**HEMICELLULASES (DEBRANCHING AND OLIGOSACCHARIDE-DEGRADING ENZYMES)**														
CBM11	pfam03425	6.7	8.7	7.7	6.9	8.2	23.5	26.7	26.7	25.0	0.0	0.1	0.0	0.6
CBM13	–	0.0	0.0	0.0	0.0	4.1	0.0	0.0	6.7	0.0	0.0	3.7	1.6	10.7
CBM22	–	0.0	0.0	0.0	0.0	0.0	0.0	0.0	0.0	0.0	15.4	6.2	0.4	4.1
CBM32	–	1.7	0.0	0.0	1.7	0.0	5.9	6.7	6.7	37.5	0.0	3.2	3.4	18.4
CBM35	–	23.3	17.4	19.2	27.6	16.3	0.0	0.0	6.7	0.0	0.0	3.7	0.7	7.6
CBM61	–	1.7	0.0	0.0	0.0	0.0	0.0	0.0	0.0	0.0	0.0	1.6	0.4	5.4
CBM67	–	1.7	2.2	1.9	1.7	0.0	0.0	0.0	0.0	0.0	0.0	0.8	1.1	8.4
Subtotals (%)		35.0	28.3	28.8	37.9	28.6	29.4	33.3	46.7	62.5	15.4	19.3	7.6	
**CELLULASES/HEMICELLULASES**														
CBM6	pfam03422	41.7	45.7	46.2	37.9	44.9	5.9	6.7	6.7	0.0	15.4	4.9	0.7	8.2
CBM9	pfam02018	0.0	0.0	0.0	0.0	0.0	0.0	0.0	0.0	0.0	7.7	5.0	0.9	8.4
Subtotals (%)		41.7	45.7	46.2	37.9	44.9	5.9	6.7	6.7	0.0	23.1	9.9	1.6	
**OTHERS**														
CBM48	pfam02922	1.7	2.2	1.9	1.7	2.0	5.9	13.3	6.7	12.5	7.7	7.5	23.0	65.9
CBM66	–	0.0	0.0	0.0	0.0	0.0	0.0	0.0	0.0	0.0	7.7	1.1	1.1	7.6
Subtotals (%)		1.7	2.2	1.9	1.7	2.0	5.9	13.3	6.7	12.5	15.4	8.6	24.1	
Total estimated CBM		60	46	52	58	49	17	15	15	8	13			
Total estimated genes		2871	2754	3008	2906	2344	3391	3347	2773	2304	2321			
CBM to estimated genes (%)		2.1	1.7	1.7	2.0	2.1	0.5	0.4	0.5	0.3	0.6	1.4^b^	0.3^c^	

a *Based on Koeck et al. (2014)*.

b *Average of 52 CBMs to total genes across 35 cellulolytic bacteria genomes*.

c *Average of 52 CBMs to total genes across 2038 bacterial species*.

As previously reported for *F. succinogenes* (Suen et al., 2011), no clostridial-like cohesin or dockerin-like modules were identified in any of the Fibrobacteres genomes, indicative of an absence of cellulosomes in this lineage. Two other putative cellulose binding proteins have been reported in *F. succinogenes;* TIGR02145 and 02148 (Morrison et al., 2009; Suen et al., 2011). TIGR02145 is a domain of ~175 to 200 amino acids with an inferred extracytoplasmic location, and has been suggested to be a possible cohesin analog (Warnecke et al., 2007). It is present in high copy number in all of the Fibrobacteres genomes (17 to 119 copies) with the exception of *C. alkaliphilus*. TIGR02148 is a fibro-slime domain-containing protein originally identified in the *F. succinogenes* genome and implicated in adherence to plant biomass (Toyoda et al., 2009). We found this protein family to be present in all Fibrobacteres genomes, again with the exception of *C. alkaliphilus* (Table 4). Therefore, these putative adhesion proteins are not only distinctive of *F. succinogenes*, but of the Fibrobacteres phylum as a whole. A phylogenetic reconstruction of the fibro-slime protein family indicates multiple duplication events in the class *Fibrobacteria* resulting in up to 10 copies per genome (Supplementary Figure 2; Table 4). Interestingly, one of the two fibro-slime proteins identified in the termite *Chitinivibrionia* genome, MC_77, contains a flagellar domain (*flgD*) suggesting that polymer attachment in this species may be flagella-mediated. Type IV pili are known to facilitate attachment of *F. succinogenes* cells to cellulose (Qi et al., 2007) and Gram negative cells to chitin (Li et al., 2003; Giltner et al., 2012). All Fibrobacteres genomes contain the necessary genes for synthesis of Type IV pili (Table 4) suggesting that this may be a widespread auxiliary mechanism used by members of this phylum to attach to polymers, and perhaps, to facilitate a “twitching” motility phenotype.

**Table 4:** **Inventory of accessory attachment genes for polymer hydrolysis identified in the Fibrobacteres genomes, organized by functional category**.

**CAZy family**	**Function ID**	**Fibrobacteria**								**Chitinivibrionia**		**Cellulolytic bacteria (35 genomes^a^)**	**Bacterial average across 3454 sp.^b^**
		**Fibrobacteraceae**					**Fibromonadaceae**						
		***F. succino-genes* S85**	**AD_80**	**AD_111**	**SR_36**	**AD_312**	**IN01_31**	**IN01_221**	**IN01_307**	***C. alkali- philus* ACht1**	**MC_77**	**Avg**	
**OTHER CELLULOSE-BINDING PROTEINS**													
Fib_succ_major	TIGR02145	54	65	72	96	17	119	116	70	0	41	0.0	0.1
Fibro-slime protein	TIGR02148	10	7	7	8	5	3	1	6	0	2	0.3	0.2
**TYPE IV PILIN GENES**													
Type IV pilin N-term methylation site PilA	pfam13544	3	3	2	3	3	4	2	2	6	2	2.2	2.7
Type IV pilus assembly protein PilB	K02652	1	0	1	1	2	1	1	1	2	3	0.9	0.4
Type IV pilus assembly protein PilC	K02653	1	1	1	1	1	1	1	1	2	1	0.8	0.4
Leader peptidase (prepilin peptidase) / N-methyltransferase [EC:3.4.23.43 2.1.1.-]	K02654	1	1	1	1	1	1	1	1	1	1	1.1	0.5
Type IV pilus assembly protein PilE	K02655	0	0	0	0	0	0	0	0	2	0	0.1	0.2
Type IV pilus assembly protein PilM	K02662	1	1	1	1	1	1	1	1	1	1	0.7	0.3
Type IV pilus assembly protein PilN	K02663	1	1	1	1	1	1	1	1	1	1	0.4	0.3
Type IV pilus assembly protein PilO	K02664	1	1	1	1	1	1	1	1	1	1	0.3	0.2
Type IV pilus assembly protein PilQ	K02666	2	2	2	2	2	1	1	1	1	1	0.1	0.2
Two-component system, NtrC family, response regulator PilR	K02667	0	0	0	0	1	0	0	0	0	0	0.1	0.1
Twitching motility protein PilT	K02669	3	2	3	2	3	2	2	2	3	2	0.9	0.5

a *Based on Koeck et al. (2014)*.

b *Average across 3454 bacterial genomes on IMG*.

#### Fermentative metabolism and respiration

**Figure 3:**
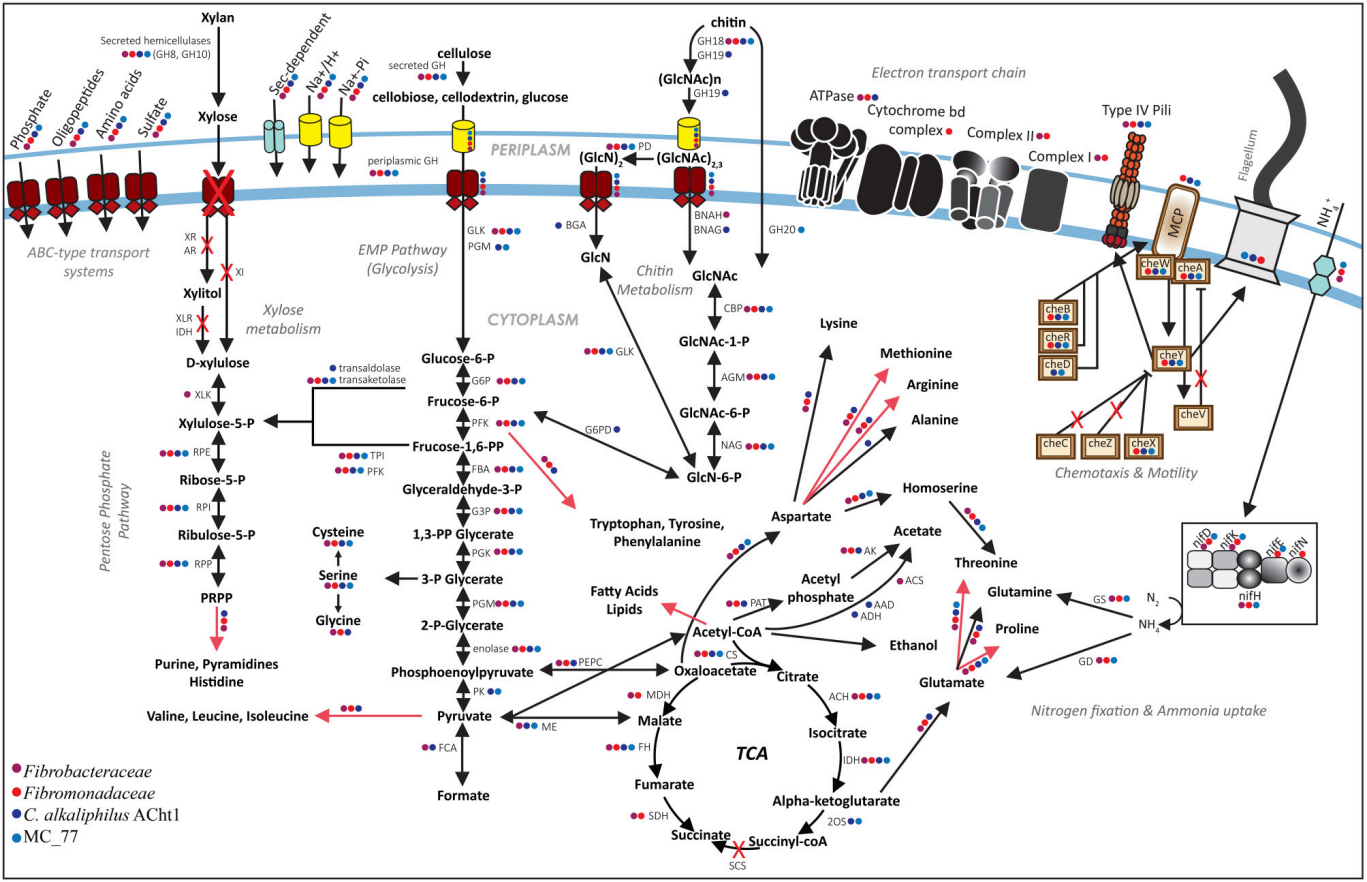
**Composite metabolic reconstruction of members of the phylum Fibrobacteres**. Presence of genes and pathways in a given lineage is indicated by colored dots (legend at lower left). Steps in metabolic pathways absent in all investigated Fibrobacteres genomes are indicated by red crosses. Multistep reactions are shown by red arrows. Abbreviations are described in Table S3.

We expected that fermentation of sugars resulting from polymer hydrolysis would be the primary metabolism in the Fibrobacteres based on the obligate fermentative phenotype of *F. succinogenes* (Suen et al., 2011) and *C. alkaliphilus* (Sorokin et al., 2014). Metabolic reconstruction indicates that all Fibrobacteres genomes have the potential to utilize glucose via the Embden-Meyerhof pathway (EMP) and pentose phosphate pathway (PPP), but not via the Entner-Doudoroff pathway which is absent (Figure 3). It has previously been noted that *F. succinogenes* and *C. alkaliphilus* are unable to grow on xylan as a sole carbon source which suggests that they use their xylanases simply to expose cellulose and chitin respectively rather than using the resulting xylose as a growth substrate (Suen et al., 2011; Sorokin et al., 2014). In that context, all Fibrobacteres lack the genes encoding a xylose permease and xylose interconversions via xylulose to xylulose-5-P which could then be processed via the PPP (Figure 3), suggesting the inability to use xylose is a phylum-level trait. The ability to use chitin hydrolysis products appears to be limited to the *Chitinivibrionia* genomes. All investigated Fibrobacteres should be able to perform the initial hydrolysis of insoluble chitin to smaller soluble oligosaccharides via GH18, which can be imported into the periplasm via TonB-dependent transporters (Figure 3). However, either GH19 (*C. alkaliphilus* only) or GH20 (MC_77 only) are required to hydrolyse the soluble oligosaccharides into N-acetylglucosamine (GlcNAc) dimers or trimers, which can then be converted into fructose-6-P and enter the EMP or PPP pathways (Sorokin et al., 2014). For all studied genomes, the end products of the EMP pathway, phosphoenoylpyruvate and pyruvate, can then enter the tricarboxylic acid (TCA) cycle or the latter can be metabolized to formate, acetate or ethanol. All 10 genomes encode incomplete TCA cycles as they lack succinyl-CoA-synthase as previously noted for both *F. succinogenes* (Suen et al., 2011) and *C. alkaliphilus* (Sorokin et al., 2014). All *Fibrobacteria* also lack 2-oxoglutarate synthase and the two *Chitinivibrionia* representatives lack succinate dehydrogenase suggesting succinate and fumarate are end products of the reductive arm of the TCA cycle for these classes, respectively (Figure 3). Succinate is a major fermentative end product of *F. succinogenes* (Suen et al., 2011), but fumarate does not accumulate as a fermentation product of *C. alkaliphilus* (Sorokin et al., 2014).

All of the investigated *Fibrobacteraceae* and *Chitinivibrionia* genomes lack major components of the electron transport chain (ETC) and are incapable of growth via respiration, which is consistent with previous reports that their characterized representatives are obligate anaerobes (Suen et al., 2011; Sorokin et al., 2014). By contrast, the *Fibromonadaceae* genomes encode an ETC comprising complexes I and II, cytochrome bd and an ATP synthase, which should be able to perform some form of electron-transport linked phosphorylation (Figure 3; Supplementary Table 3). The cytochrome bd complex in other bacteria functions under low oxygen conditions (Borisov et al., 2011), which is consistent with the termite hindgut habitat from which the *Fibromonadaceae* genomes were obtained. Due to its small size, the termite hindgut is only anoxic in the central region and has microoxic peripheries (Brune et al., 1995). To investigate the origins of the *Fibromonadaceae* ETC, we inferred phylogenetic trees from the most conserved components (bd complex), which indicate that the common ancestor of the family had an ETC which is distantly related to other phyla and unlikely to be the result of a recent lateral transfer (Supplementary Figure 3). Other lineages within the Fibrobacteres, currently lacking genomic representation (Figure 1B), may also have ETCs, which if present, will help to shed light on the ancestry of respiration in this phylum. All Fibrobacteres genomes, with the exception of MC_77, encode enzymes to counter oxidative stress including thioredoxin reductase and superoxide dismutase, but not catalase (Supplementary Table 3). The apparent absence of antioxidant enzymes in MC_77 may be an artifact of the lower estimated completeness (73.3%) of this genome.

#### Nitrogen and ammonia metabolism

Lignocellulosic biomass is nitrogen limited and a poor source of amino acids, vitamins and their precursors (Brune, 2014). Metabolic reconstruction revealed a sporadic distribution of core nitrogen fixing genes (*nifH, nifD*, and *nifK*) amongst the Fibrobacteres representatives (Figures 1, 3, Supplementary Table 3), suggesting a history of gain and loss by lateral gene transfer as previously noted more generally for nitrogen fixation (Boucher et al., 2003). We created phylogenetic trees for NifD and NifK and infer that the genes encoding these proteins were recently and independently acquired by the *Fibrobacteraceae, Fibromonadaceae*, and *Chitinivibrionia* from different Firmicutes donors (Supplementary Figure 4). Genes immediately flanking the *nif* genes were conserved in each family supporting lateral acquisition (Supplementary Figure 5). Our data are therefore not consistent with the idea of an early acquisition of nitrogen-fixing genes in the Fibrobacteres (Suen et al., 2011), but rather suggest a patchy history of recent gain and loss in habitats where nitrogen-fixing genes are present in numerous other community members providing the opportunity for lateral transfer (Warnecke et al., 2007; Brulc et al., 2009; He et al., 2013). Whether the *nif* genes are functionally active is debatable as *F. succinogenes*, which contains only four *nif* genes (3 core; *nifH,D,K*), has not been shown to be capable of nitrogen fixation (Suen et al., 2011). If any of the Fibrobacteres are capable of nitrogen fixation, they have amongst the lowest recorded number of subunits (3 to 9) for an active nitrogenase (Wang et al., 2013). By contrast, all members of the Fibrobacteres have ammonia uptake and assimilation genes (Supplementary Table 3) which may supply their nitrogen requirements (Matheron et al., 1999; Suen et al., 2011; He et al., 2013). All 10 of the Fibrobacteres genomes have the potential to synthesize most of their own amino acids and cofactors (Figure 3; Supplementary Table 3), including the gut symbionts, suggesting that they are not dependent on other organisms or host diet for most of their nutritional requirements.

#### Motility and chemotaxis

Fibrobacteres have been defined as non-motile bacteria based on their only characterized representative genus, *Fibrobacter* (Ransom-Jones et al., 2012; Jewell et al., 2013). However, far from being a phylum-level trait, all investigated members of the *Fibromonadaceae* and *Chitinivibrionia* encode numerous flagellar and associated chemotaxis genes (Figure 3 and Supplementary Figure 6; Supplementary Table 3), which is consistent with the direct observation of a polar flagellum in *C. alkaliphilus* (Sorokin et al., 2014). Methyl-accepting chemotaxis proteins were notably more abundant in the *Fibromonadaceae* and MC_77 genomes than in *C. alkaliphilus* (Supplementary Figure 7) despite the closer phylogenetic relationship of MC_77 to *C. alkaliphilus*. This may reflect habitat differences since *Fibromonadaceae* and MC_77 reside in termite guts which have complex chemical milieus and steep chemical gradients likely requiring motile microorganisms to respond to a wider range of environmental cues than *C. alkaliphilus* in a hypersaline soda lake. Putative sensory hydrogenases were identified in members of both the *Fibromonadaceae* and MC_77 (Supplementary Figure 7), which are hypothesized to allow these bacteria to orient themselves to steep hydrogen gradients present in the termite gut (Warnecke et al., 2007). The absence of flagella and chemotaxis previously reported for *F. succinogenes* (Suen et al., 2011) appears to be a family-level trait in the *Fibrobacteraceae* (Figure 3 and Supplementary Figure 8; Supplementary Table 3). Phylogenetic analysis of several core flagellar genes (Liu and Ochman, 2007) suggest that motility was vertically inherited from a common Fibrobacteres ancestor and subsequently lost in the *Fibrobacteraceae* lineage (Supplementary Figure 8). Since, most members of this family have adapted to life in the herbivore gut, flagella-enabled chemotaxis and motility may have been no longer required due to an abundance of degradable substrates and mixing of contents provided by the host animal. Further genomic representation of the phylum will be required to determine if other lineages within the Fibrobacteres have similarly lost motility genes.

## Conclusion

In this study, we have substantially expanded the phylogenomic representation of the Fibrobacteres and TG3 lineages by obtaining eight draft genomes of environmental populations from termite guts, anaerobic cellulose-fed digester, and a sheep rumen. We propose that TG3 should be amalgamated with the Fibrobacteres phylum because the two lineages are robustly monophyletic in concatenated marker gene trees, and because they share a number of key traits. These include polymer hydrolysis which appears to be a unifying feature of the phylum, reflected by environmental distribution in habitats in which polymer hydrolysis plays a major role. As with *F. succinogenes*, all Fibrobacteres representatives have xylanases, but lack the genes necessary to metabolize xylan degradation products for energy transduction. In contrast to previous suppositions largely based on characteristics of the genus *Fibrobacter*, we infer that not all members of the Fibrobacteres are strictly anaerobic as some have respiratory chains, and most appear to be motile. Members of the family *Fibromonadaceae* have low oxygen bd cytochromes allowing them to respire in microaerophilic conditions, and flagella-mediated motility is inferred to be an ancestral trait in the phylum having being lost from the family *Fibrobacteraceae*. Nitrogen fixing genes are sporadically distributed across the phylum and appear to have been obtained by multiple independent lateral transfers, whereas salvaging of fixed nitrogen from ammonia is inferred to be a more general trait. The eight population genomes described in the present study form an improved basis for further investigations into the Fibrobacteres phylum.

### Description of “*Candidatus Fibromonas termitidis*”

*Fibromonas termitidis* (Fi.bro.mo'nas L. fem. n. *fibra*, fiber or filament in plants or animals; Gr. fem. n. *monas*, a unit, monad. ter.- mi'ti.dis. L. n. *tarmes, tarmit*- (L.L.var. *termes, termit*-) worm that eats wood; M.L. adj. *termitidis* pertaining to the termite). Not cultivated. Inferred to be Gram-negative, motile, containing an ETC, and able to use cellulose as a primary growth substrate. Represented by population genome IN01_221 (acc. no. LIUG00000000) obtained from metagenomes of whole gut samples of the higher termite, *Microcerotermes* (acc. no. KJ907817).

### Description of *Fibromonadaceae* (fam. nov.)

The description is the same as for the genus *Fibromonas*; *-aceae* ending to denote an family. Type genus: *Candidatus* Fibromonas.

### Description of *Fibromonadales* (ord. nov.)

The description is the same as for the genus *Fibromonas*; *-ales* ending to denote an order. Type family: *Fibromonadaceae* fam. nov.

### Emended description of the phylum Fibrobacteres garrity and holt 2001 (Spain et al., 2010)

The phylum Fibrobacteres is a deep-branching lineage of the Bacteria. On the basis of comparative sequence analysis of isolate and environmental genomes, the phylum comprises at least two classes; *Fibrobacteria* and *Chitinivibrionia*, and three orders; *Fibrobacterales, Fibromonadales*, and *Chitinivibrionales*. Gram-negative, polymer-degrading bacteria.

### Conflict of interest statement

The authors declare that the research was conducted in the absence of any commercial or financial relationships that could be construed as a potential conflict of interest.
