# Supplementary material for: A Phylogenomic Analysis of the Bacterial Phylum Fibrobacteres
Source: Front Microbiol. 2016 Jan 7;6:1469. doi: 10.3389/fmicb.2015.01469 (PMC4704652; doi:10.3389/fmicb.2015.01469)
Supplement: Supplementary file 2 [file Presentation1.PDF]

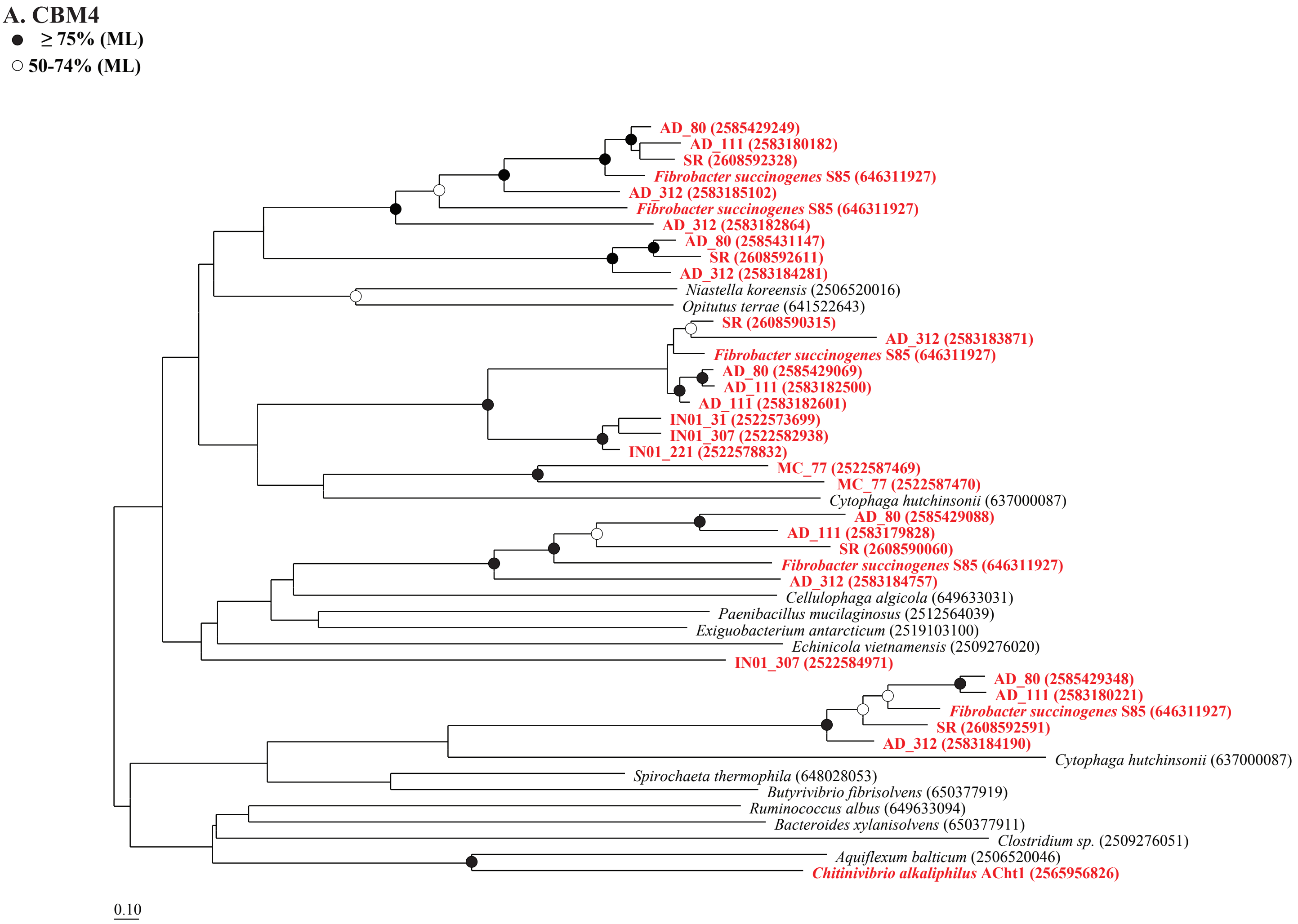

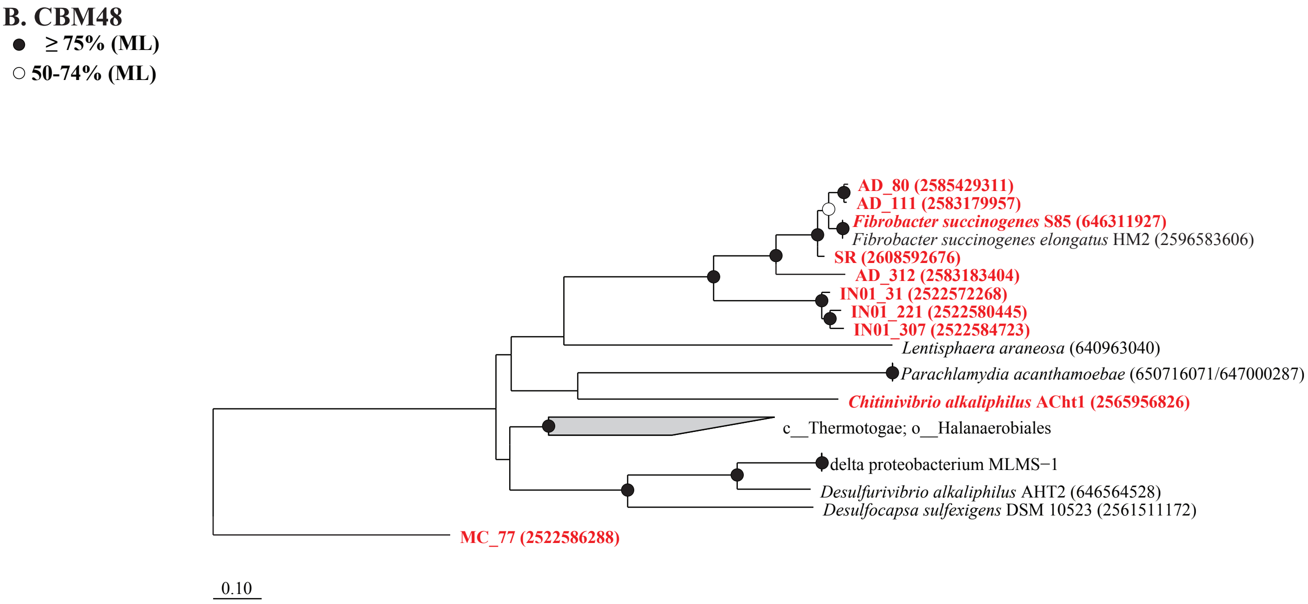

Supplementary Figure 1. A maximum likelihood phylogenetic analysis of two CBM families; A) CBM4 and B) CBM46 >722aa. Carbohydrate binding module (CBM) protein sequences were aligned using MAFFT v7.221 and phylogenetic trees were constructed from 2,256 finished genomes from the IMG database (Markowitz et al., 2009) using Fasttree v2.1.7. The trees are unrooted and only the closest neighbors of the Fibrobacteres (in red) are shown with corresponding IMG IDs in brackets. Bootstrap support for interior nodes is indicated by dots according to the legend at the top left of the figure.

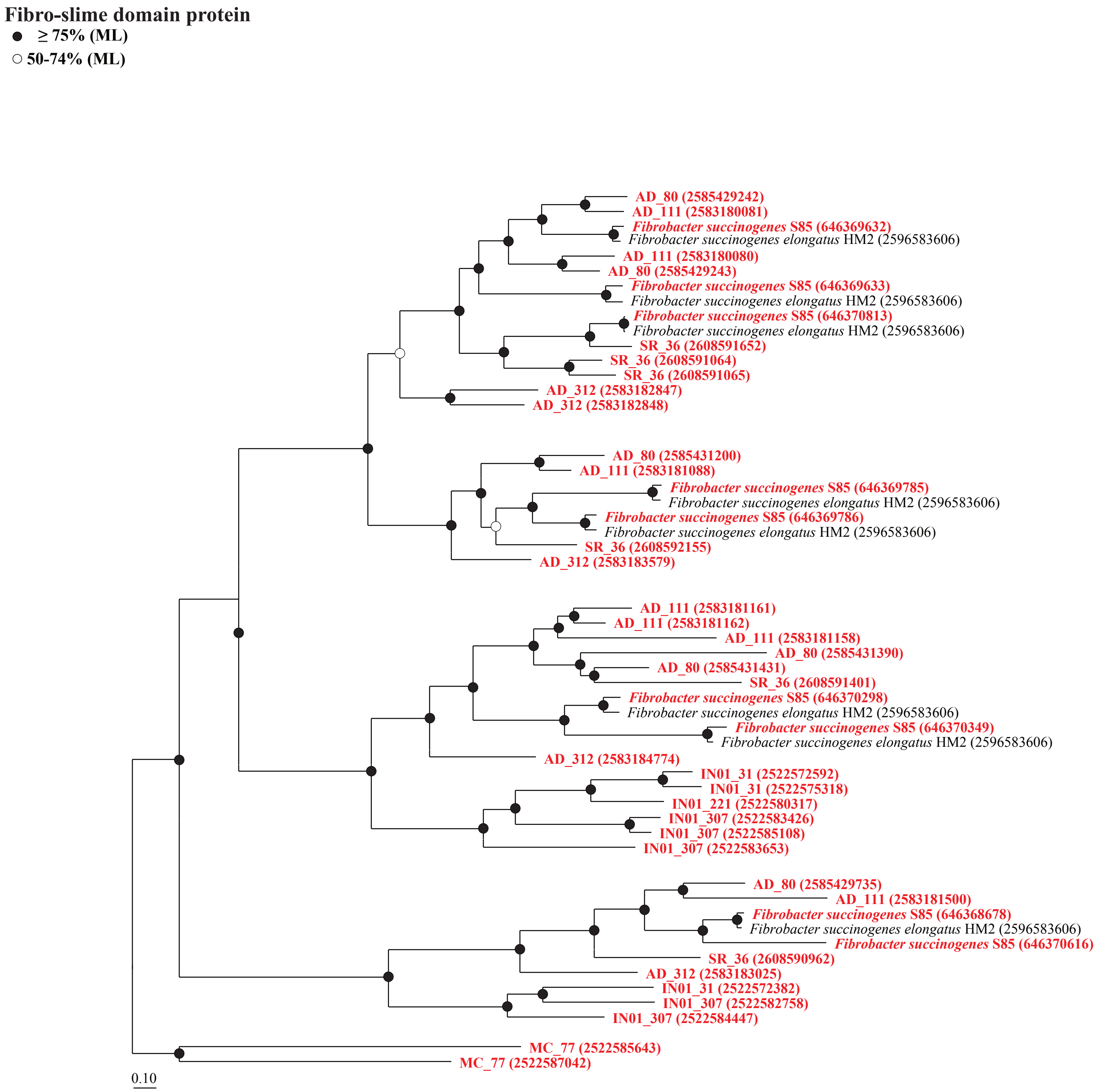

Supplementary Figure 2. A maximum likelihood phylogenetic analysis of fibro-slime domain proteins >600aa. Fibro-slime domain protein sequences were aligned using MAFFT v7.221 and phylogenetic trees were constructed from 2,256 finished genomes from the IMG database (Markowitz et al., 2009) using Fasttree v2.1.7. The tree is rooted with MC_77 and corresponding IMG IDs shown in parentheses. Bootstrap support for interior nodes is indicated by dots according to the legend at the top left of the figure.

A. Cytochrome bd-I ubiquinol oxidase subunit 1

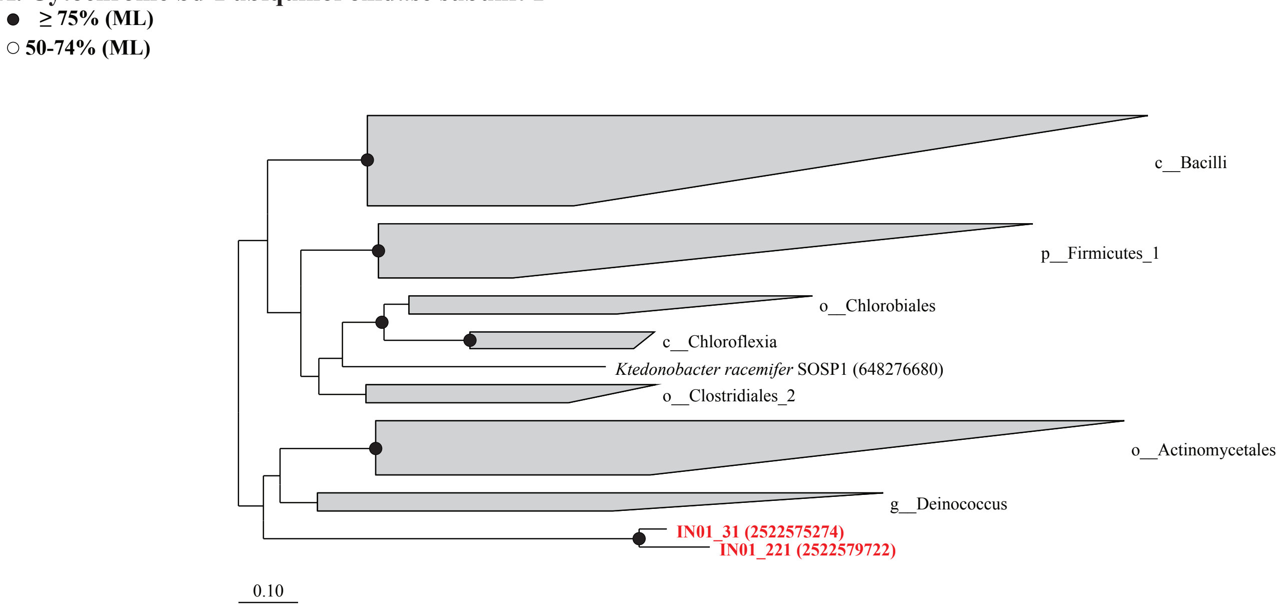

B. Cytochrome bd-I ubiquinol oxidase subunit 2

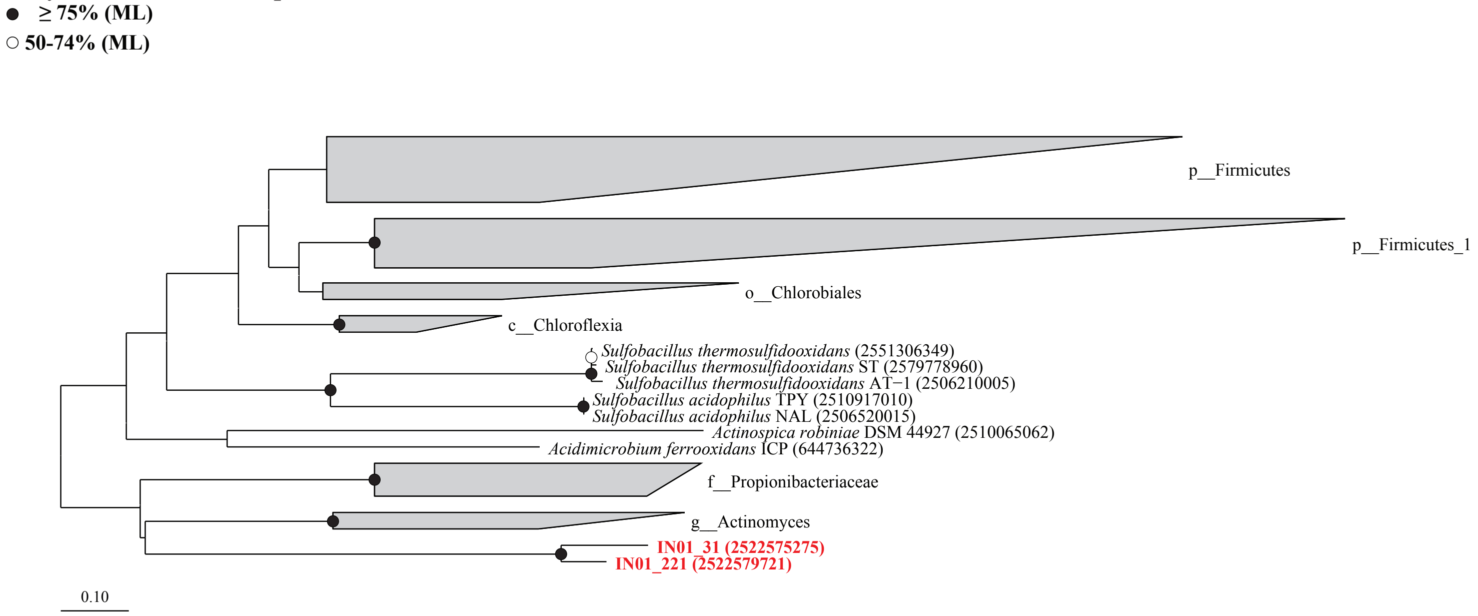

C. ABC-type transport system involved in cytochrome bd biosynthesis, fused ATPase and permease components

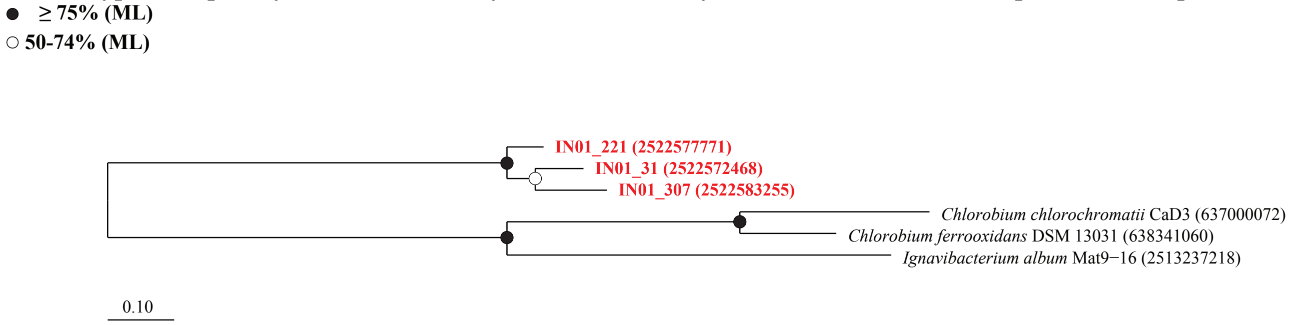

Supplementary Figure 3. A maximum likelihood phylogenetic analysis of proteins encoding cytochrome bd-I ubiquinol oxidase; A) subunit 1 and B) subunit 2 >341aa. Cytochrome bd-I ubiquinol oxidase protein sequences were aligned using MAFFT v7.221 and phylogenetic trees were constructed from 2,256 finished genomes from the IMG database (Markowitz et al., 2009) using Fasttree v2.1.7. The trees are unrooted and only the closest neighbors of the Fibrobacteres (in red) are shown with corresponding IMG IDs in brackets. Bootstrap support for interior nodes is indicated by dots according to the legend at the top left of the figure.

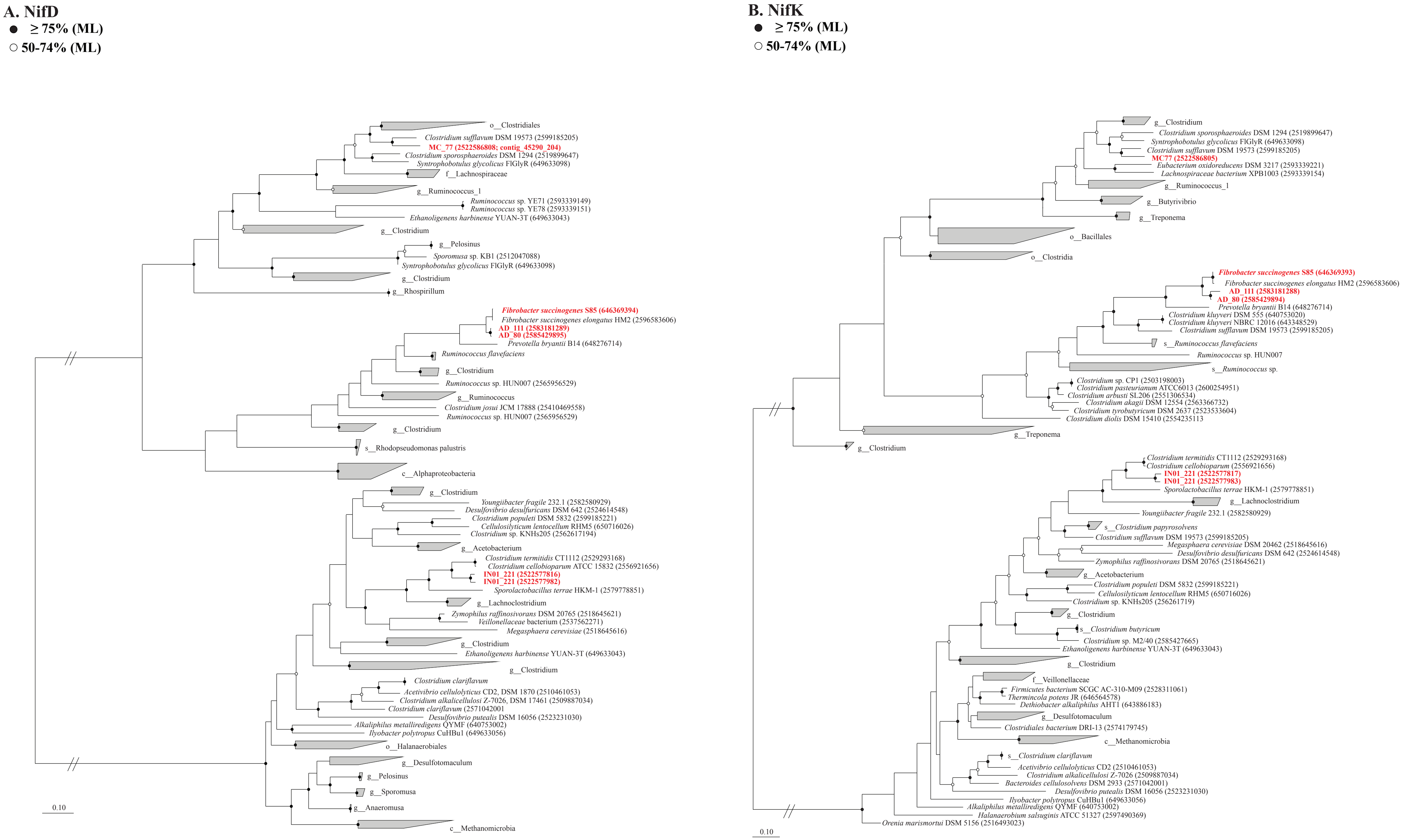

Supplementary Figure 4. A maximum likelihood phylogenetic analysis of protein encoding nitrogenase; A) NifD and B) NifK >458aa. Nitrogenase protein sequences were aligned using MAFFT v7.221 and phylogenetic trees were constructed from 2,256 finished genomes from the IMG database (Markowitz et al., 2009) using Fasttree v2.1.7. The trees are unrooted and only the closest neighbors of the Fibrobacteres (in red) are shown with corresponding IMG IDs in brackets. Bootstrap support for interior nodes is indicated by dots according to the legend at the top left of the figure.

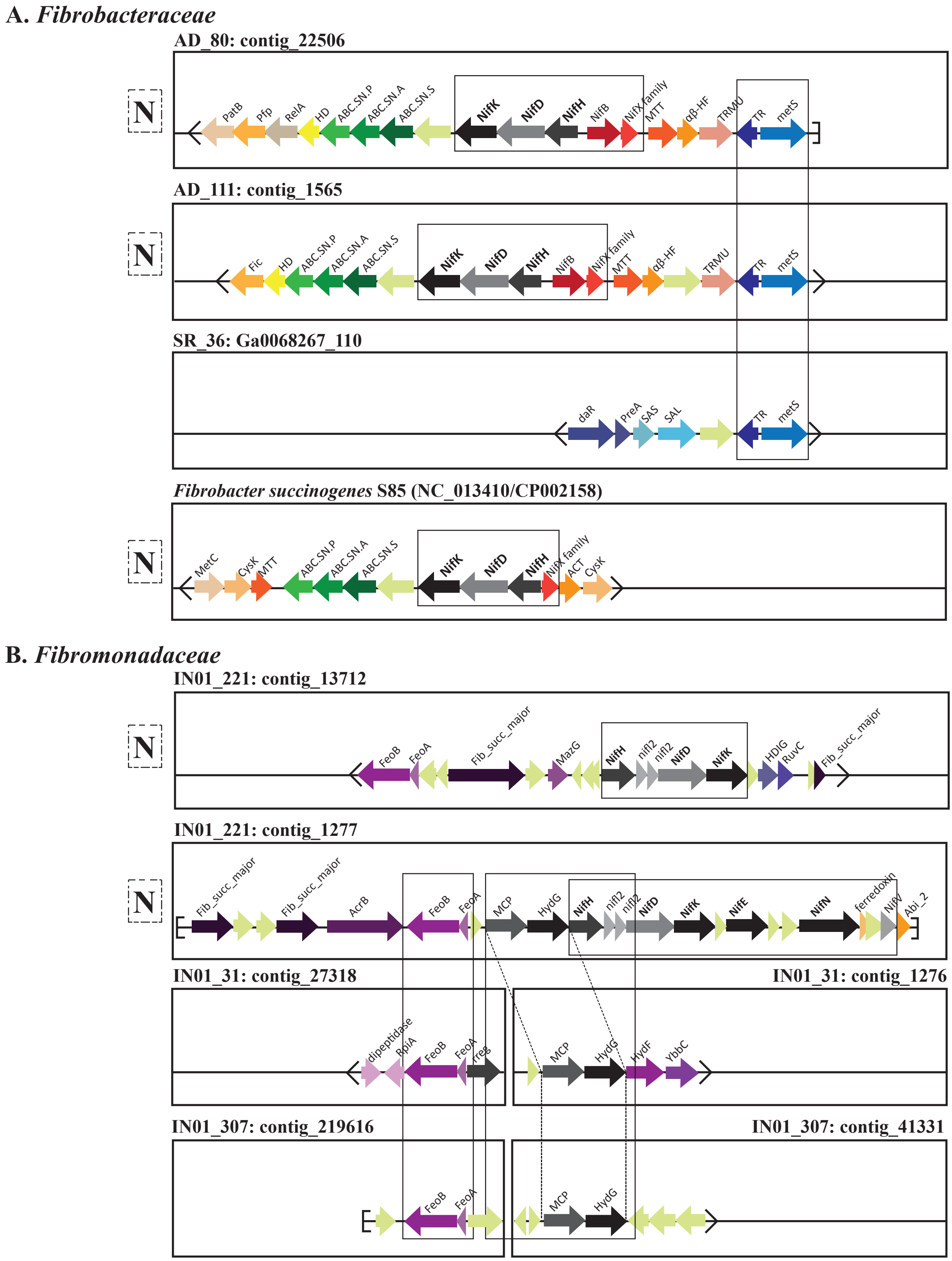

Supplementary Figure 5. Gene neighborhoods of nitrogen fixing genes in the families A) Fibrobacteraceae and B) Fibromonadaceae. Genomes containing nif genes are indicated by dotted boxed Ns to the left of the figure. Colors indicate orthologous gene families and syntenous blocks of genes are highlighted by boxing; horizontal boxes denote nif genes and vertical boxes are common flanking region.

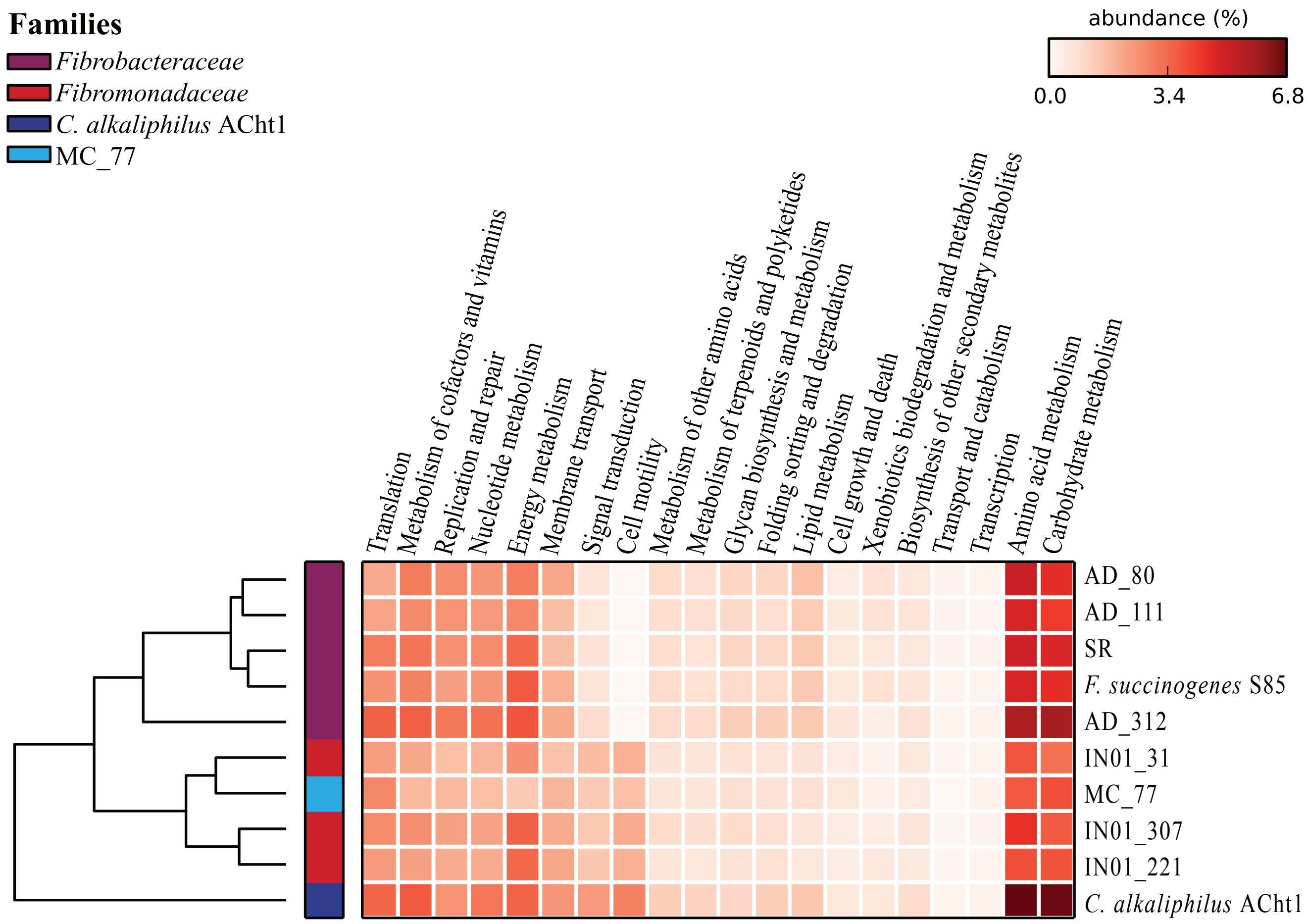

Supplementary Figure 6. Relative abundance of KEGG ortholog (KO) functional categories across the investigated Fibrobacteres genomes. Genomes are ordered by similarity of relative abundance patterns.

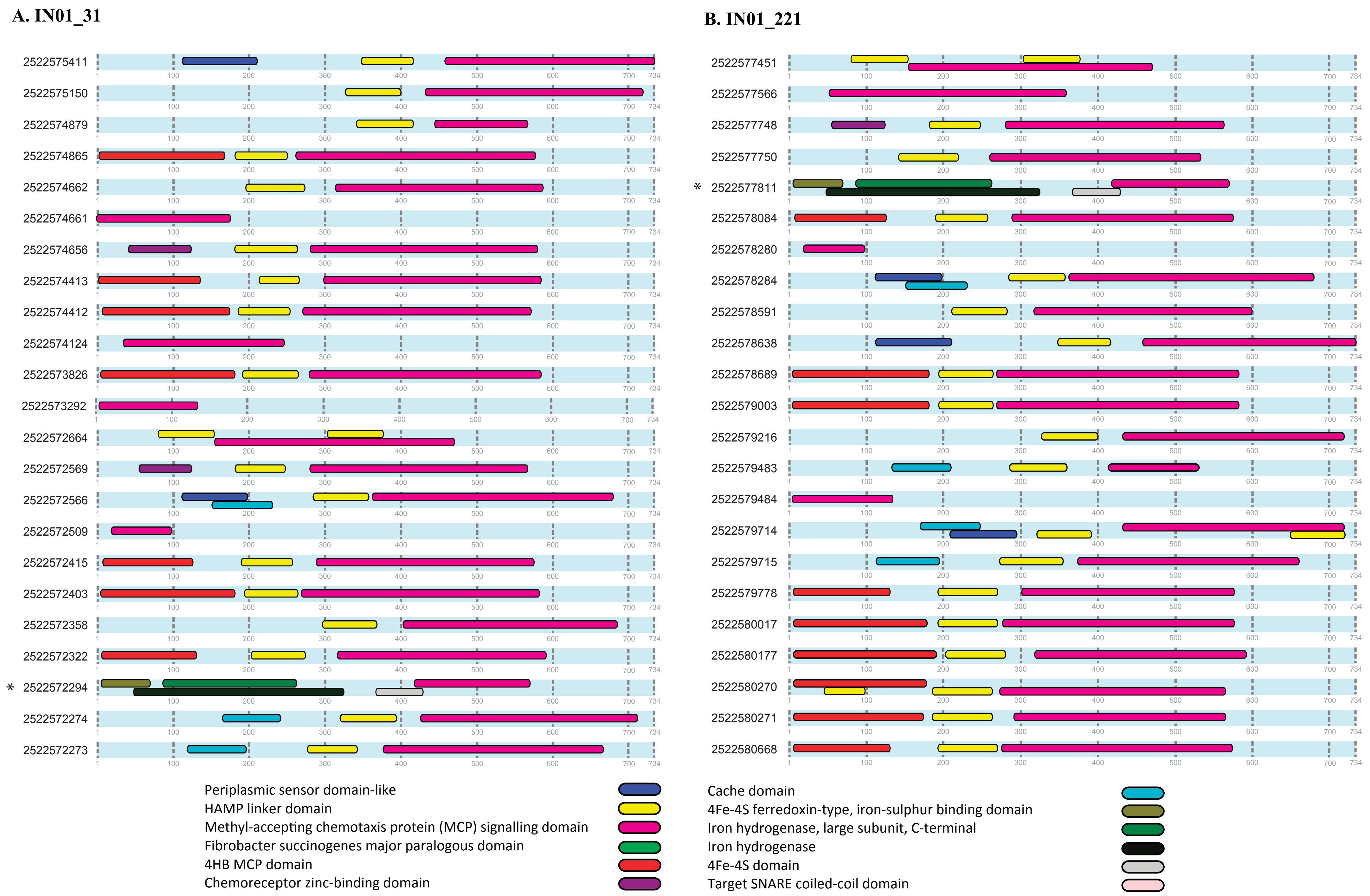

Supplementary Figure 7. Methyl-accepting chemotaxis proteins (MCP) encoded in the genomes of family Fibromonadaceae (panels A-C) and class Chitinivibrionia (panels D-E) predicted with InterProScan5 (Jones et al., 2014). Protein domains are shown as colored blocks. Putative senosry hydrogenases are indicated with asterisks.

C. IN01_307

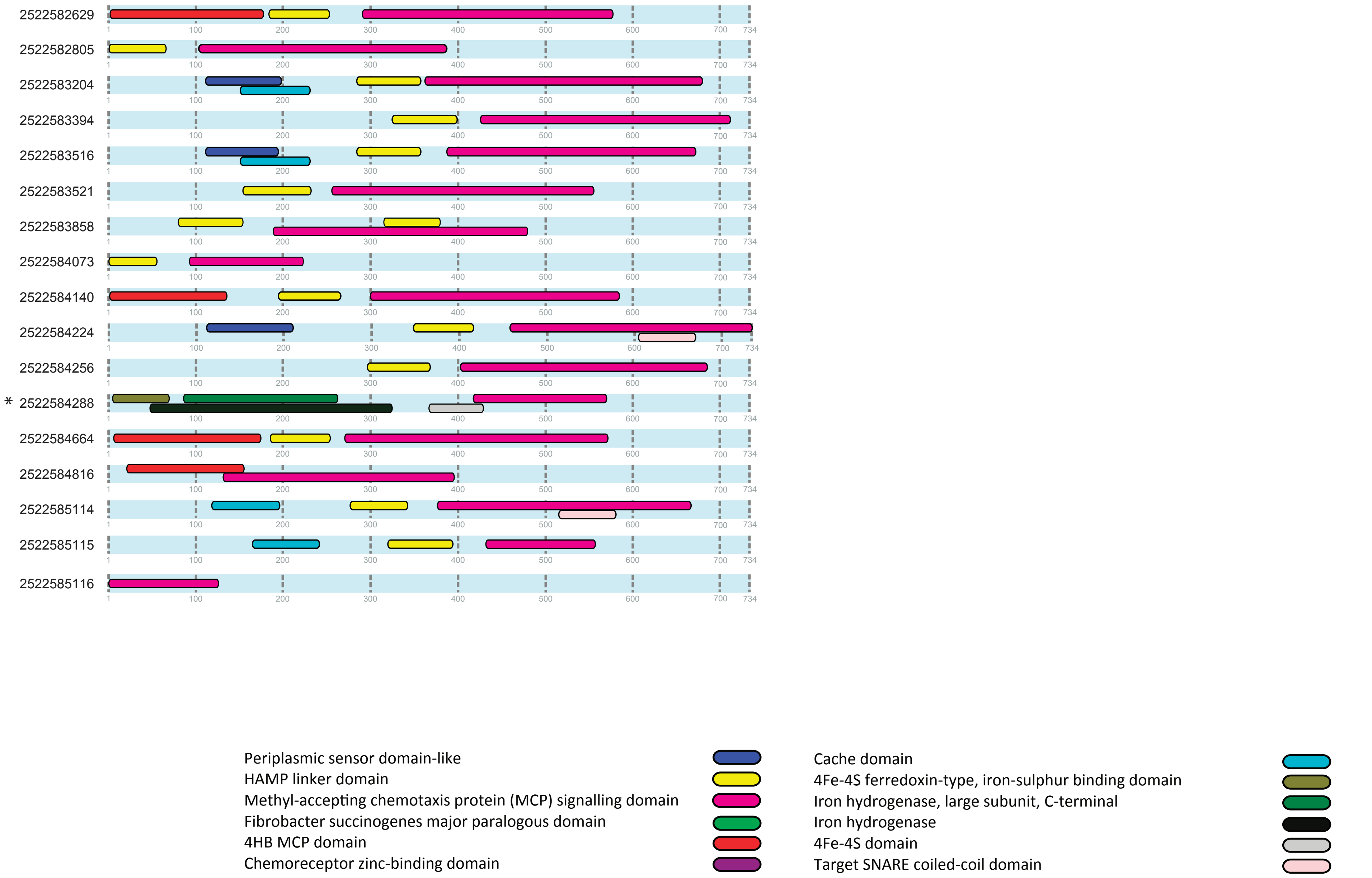

Supplementary Figure 7. Methyl-accepting chemotaxis proteins (MCP) encoded in the genomes of family Fibromonadaceae (panels A-C) and class Chitinivibrionia (panels D-E) predicted with InterProScan5 (Jones et al., 2014). Protein domains are shown as colored blocks. Putative senosry hydrogenases are indicated with asterisks.

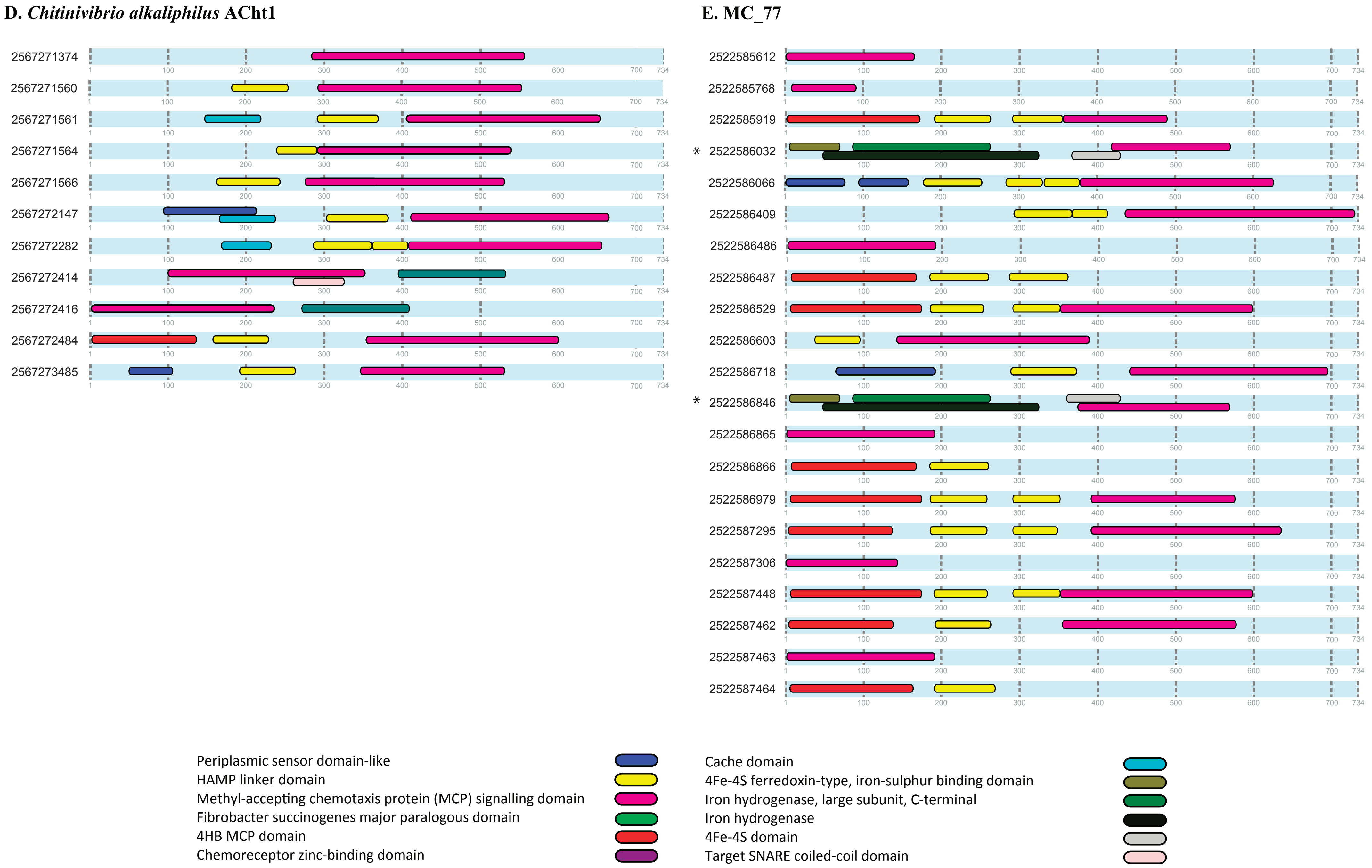

Supplementary Figure 7. Methyl-accepting chemotaxis proteins (MCP) encoded in the genomes of family Fibromonadaceae (panels A-C) and class Chitinivibrionia (panels D-E) predicted with InterProScan5 (Jones et al., 2014). Protein domains are shown as colored blocks. Putative senosry hydrogenases are indicated with asterisks.

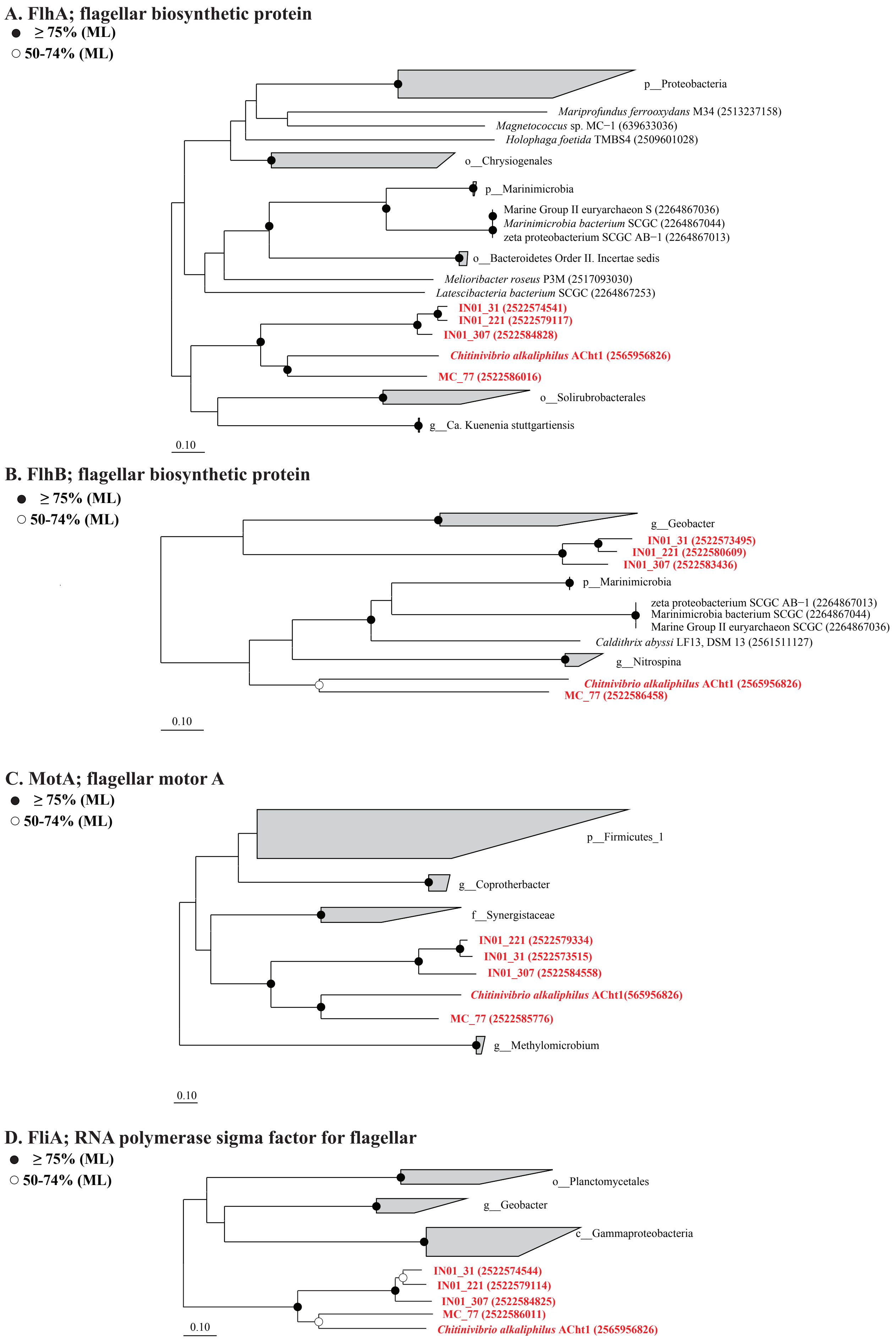

Supplementary Figure 8. A maximum likelihood phylogenetic analysis of protein encoding flagellar genes; A) Flagellar biosynthetic proteins (FlhA), B) Flagellar motor proteins (FlhB), C) RNA polymerase sigma factor for flagellar (MotA), D) Flagellar hook-basal body complex (FliA). Flagellar protein sequences were aligned using MAFFT v7.221 and phylogenetic trees were constructed from 2,256 finished genomes from the IMG database (Markowitz et al., 2009) using Fasttree v2.1.7. The trees are unrooted and only the closest neighbors of the Fibrobacteres (in red) are shown with corresponding IMG IDs in brackets. Bootstrap support for interior nodes is indicated by dots according to the legend at the top left of the figure. The length of amino acid sequences ranges from 339 to 957.

E. FliE; flagellar hook-basal body complex

● ≥ 75% (ML)
○ 50-74% (ML)

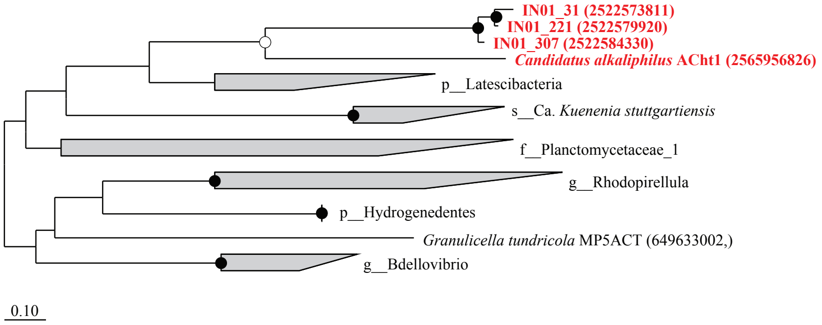

F. FliP; flagellar biosynthetic protein

● ≥ 75% (ML)
○ 50-74% (ML)

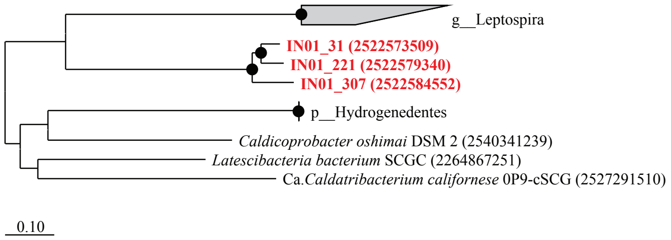

G. FliS; flagellar protein

● ≥ 75% (ML)
○ 50-74% (ML)

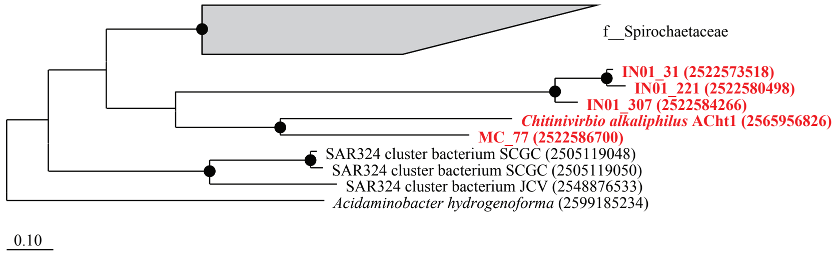

H. FlgK; flagellar hook-associated protein 1

● ≥ 75% (ML)
○ 50-74% (ML)

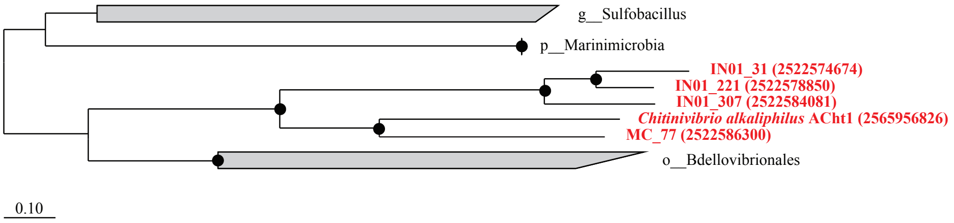

I. FlgI; flagellar P-ring protein precursor

● ≥ 75% (ML)
○ 50-74% (ML)

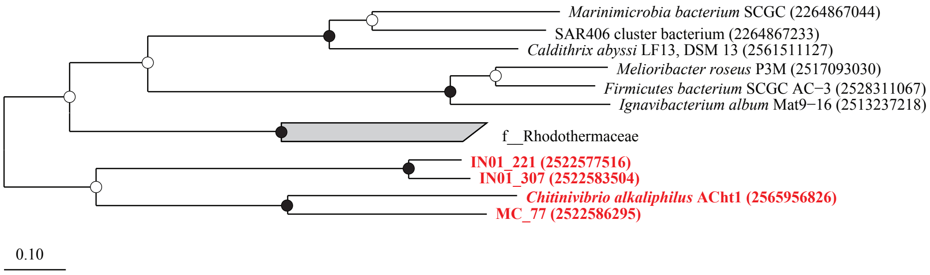

Supplementary Figure 8. A maximum likelihood phylogenetic analysis of protein encoding flagellar genes; E) Flagellar biosynthetic protein (FliE), F) Flagellar protein (FliP), G) Flagelar hook-associated protein I (FliS), H) Flagellar P-ring protein precursor (FlgK), I) Flagellar L-ring protein precursor (FlgI). Flagellar protein sequences were aligned using MAFFT v7.221 and phylogenetic trees were constructed from 2,256 finished genomes from the IMG database (Markowitz et al., 2009) using Fasttree v2.1.7. The trees are unrooted and only the closest neighbors of the Fibrobacteres (in red) are shown with corresponding IMG IDs in brackets. Bootstrap support for interior nodes is indicated by dots according to the legend at the top left of the figure.

J. FlgH; flagellar L-ring protein precursor

Bootstrap values

● ≥75

○ 50-74

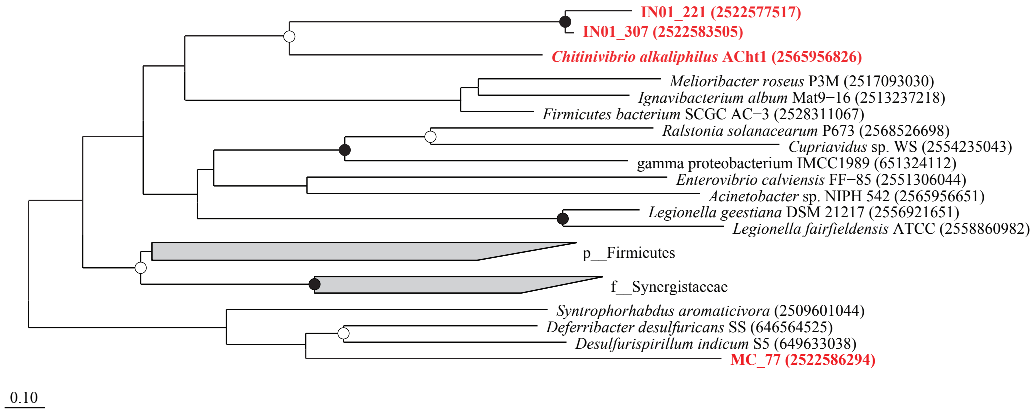

K. FlgC; flagellar basal-body rod protein

Bootstrap values

● ≥75

○ 50-74

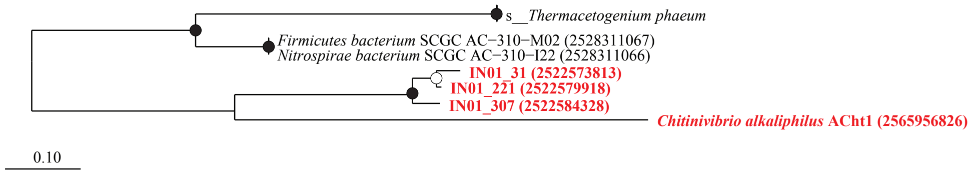

Supplementary Figure 8. A maximum likelihood phylogenetic analysis of protein encoding flagellar genes; J) flagellar L-ring protein precursor (FlgH), K) flagellar basal-body rod protein. Flagellar protein sequences were aligned using MAFFT v7.221 and phylogenetic trees were constructed from 2,256 finished genomes from the IMG database (Markowitz et al., 2009) using Fasttree v2.1.7. The trees are unrooted and only the closest neighbors of the Fibrobacteres (in red) are shown with corresponding IMG IDs in brackets. Bootstrap support for interior nodes is indicated by dots according to the legend at the top left of the figure.
